# A survey on performance evaluation of artificial intelligence algorithms for improving IoT security systems

**DOI:** 10.1038/s41598-023-46640-9

**Published:** 2023-12-01

**Authors:** Hind Meziane, Noura Ouerdi

**Affiliations:** LACSA Laboratory, Faculty of Sciences (FSO), Mohammed First University (UMP), Oujda, Morocco

**Keywords:** Computer science, Information technology

## Abstract

Security is an important field in the Internet of Things (IoT) systems. The IoT and security are topical domains. Because it was obtained 35,077 document results from the Scopus database. Hence, the AI (Artificial Intelligence) has proven its efficiency in several domains including security, digital marketing, healthcare, big data, industry, education, robotic, and entertainment. Thus, the contribution of AI to the security of IoT systems has become a huge breakthrough. This contribution adopts the artificial intelligence (AI) as a base solution for the IoT security systems. Two different subsets of AI algorithms were considered: Machine Learning (ML) and Deep Learning (DL) methods. Nevertheless, it is difficult to determine which AI method and IoT dataset are best (more suitable) for classifying and/or detecting intrusions and attacks in the IoT domain. The large number of existing publications on this phenomenon explains the need for the current state of research that covers publications on IoT security using AI methods. Thus, this study compares the results regarding AI algorithms that have been mentioned in the related works. The goal of this paper is to compare the performance assessment of the existing AI algorithms in order to choose the best algorithm as well as whether the chosen algorithm can be used for classifying or/and detecting intrusions and attacks in order to improve security in the IoT domain. This study compares these methods in term of accuracy rate. Evaluating the current state of IoT security, AI and IoT datasets is the main aim for considering our future work. After that, this paper proposes, as result, a new and general taxonomy of AI techniques for IoT security (classification and detection techniques). Finally, the obtained results from this assessment survey that was dedicated to research conducted between 2018 and 2023 were satisfactory. This paper provides a good reference for researchers and readers in the IoT domain.

## Introduction

Internet of Things or IoT aims at connecting devices or things to the Internet to collect and exchange data from the environment without any human intervention. IoT was first used by Kevin Ashton in 1999, who has also invented the term RFID (Radio-Frequency Identification)^1^. In recent years, IoT plays a pivotal role in several domains/fields in the market such as healthcare, agriculture, smart home, smart city, education, smart grids, smart business.

In recent years, AI has become an emergent technology in many domains such as security, industry, etc. Security is one of challenges that we have in the era of IoT. Thus, IoT is really a challenged domain. Since, it was obtained 35,077 document results from the Scopus database after using the search criterion ((iot OR "Internet of Things") AND security). Recently, the adoption of artificial intelligence (AI) algorithms for IoT security systems has increased. Due to the use of the search criterion ((iot OR "Internet of Things") AND security AND (ai OR "artificial intelligence")) we gotten 2557 document results from the Scopus database, indicating a big interest in IoT security and AI among academic researchers, as well as the importance of IoT security research topic which encourage the use of AI algorithms for improving IoT security systems.

Improving the IoT security system means a good choice of AI technique and IoT dataset to get good accuracy in the context of classifying and detecting potential IoT attacks. There are many methods to classify and detect IoT attacks and intrusions based on AI, among which, the intelligent ML approaches, and the DL approaches. In spite of all the tools that offer the possibility to protect the IoT systems, IoT systems are more vulnerable to attacks due to several restrictions and the enormous concerns related to this field. For this reason, the security of IoT systems must be a priority.

IoT security is one of the most important concerns. Hence, Security Information and Event Management (SIEM) is a tool/solution that monitors, detects and alerts about security events or incidents in an IT environment. SIEM systems combines security event management (SEM) with security information management (SIM). It aims at improving security awareness within an IT environment by combining SIM and SEM^2^. By gathering and analyzing data and sources related to both real-time/recent and historical security event activity, SIEM solutions improve security incident management, threat detection, and compliance^2^. Additionally, it helps enable user activity monitoring, security monitoring, and compliance. This tool provides various services, like vulnerability scanning, intrusion detection, availability checking. Nevertheless, this tool has not yet been appropriated to the IoT. On the other hand, the AI techniques and methods can be used for classifying and detecting attacks and intrusions in the IoT system. Nevertheless, it is still unclear which AI approach(es), dataset(s) are efficient and best for classifying and detecting attacks in order to improve IoT security. Furthermore, in order to build a performant intrusion detector that relies on the chosen AI model(s) for detecting, predicting and monitoring anomalies in IoT system, we first need to choose the best AI technique to solve the classification or/and detection problem and an IoT dataset which are the main focus, then we can apply the chosen AI technique to train the data to make predictions at the end.

In addition to those AI techniques, there is another new technique apart from ML (ML suffers from a lot of false positives, false negatives, errors), called XAI (Explainable Artificial Intelligence) which addresses the problem of “black box”. For example, while working with neural networks, programmers are unable to understand what is behind. i.e., for programmers, the neural networks lack visibility. Therefore, a set of AI algorithms that can be understood by users is defined by XAI. Which includes the human decision or (human factor as the decision-maker). It refers to the question “How and why does the ML model make a prediction or decision in this way?”. In order to maintain success, we need to keep the human factor and not neglect it, so we need humans to work together with AI.

### Objective of the work

The objective of the survey is the state of the art, it is an analysis of the related works (current state of research) that takes more time before reaching the most recent findings of these revolutionary concepts, i.e., the work that an author will do in 6 months, he will read it in a few minutes/hours, it is a good result. The goal of this survey is to clearly indicate what is missing, what is needed, and what needs to be done to improve IoT security as well as the directions in AI and IoT security research. It clearly describes what has been done before on the problem, and what is new will be detailed in the results section. So, this survey analyses the results and possible outcome from this new technology before integrating it. Therefore, this survey compares the performance of multiple AI methods in attack classification and anomaly or outlier detection. It performs a comprehensive state of the art on the study of AI for security in the IoT. The main goal is to outline the best AI method used for classification and/or detection of attacks in IoT environment. Indeed, a new and general taxonomy of AI based classification techniques for IoT security are also provided.

The question asked by this research is as follows *“which is the best AI technique or algorithm for improving IoT security?”* this means that which is the AI approach that achieves the best score in terms of accuracy, I proposed this study topic because it is very interesting for improving IoT security systems. The next question that can also be asked through this work is: “*can we use the chosen technique in order to classify and/or detect intrusions and attacks in the IoT security systems*?”. In the other words, which algorithm among the AI techniques and algorithms could be applied on classifying and/or detecting attacks and intrusions in order to improve the IoT security systems.

The problem that we have is “*how to choose an efficient AI model that we can use especially with the IoT security*?”. We need to understand a task(s) of application we are working on. The third research question is “*which datasets are most suitable for IoT systems*”. The originality or the added value of this contribution is as follows “There is no detailed other works in the literature that have reported or treated the questions posed through this research”. For this reason, it is imperative to choose a proper/right AI algorithm suitable for classification and/or detection in the IoT security systems, because choosing a wrong AI technique could lead to loss of effort, accuracy and effectiveness. For that, a comparison on accuracy will be made. Moreover, choosing a wrong dataset will produce incorrect results. For this reason, this paper gives a comparative analysis on the most available datasets to deduce recent and IoT-oriented datasets. Further, to increase accuracy, we need to follow/use a good classifier and a good dataset. Because, a good choice of AI method and IoT dataset leads to good results. Moreover, we cannot assume what is the best AI method to do attack classification and detection and for what dataset?

In this paper, the contribution of AI in the security of IoT systems is discussed. The aim of this paper is to carry out a comprehensive survey of AI techniques for IoT security systems. In other words, we make a comparison between the obtained results of AI techniques in terms of the accuracy rate. The objective of this performance comparison is to synthesize the most effective classification or/and detection method. Because, it is crucial to choose the right method and the right dataset for IoT security systems. On the other hand, the main idea of this research work is to collect and evaluate different results found by scientific researchers/academics/authors who have used AI in the context of classifying and/or detecting potential IoT attacks and intrusions or anomalies in order to find the best AI method taking into account the most suitable IoT dataset(s).

The paper is the only one that provides an in-depth analysis of the AI and IoT security fields. It is not about a descriptive overview. This study gives readers with more information about the best AI technique to use in classifying/detecting IoT attacks, and the data that comes from the IoT environment to achieve the best prediction results. It also gives the current state of research using AI techniques and other than AI. Further, no significant study on the best classifier or AI model(s), appropriate dataset(s) for IoT environment has been discussed. Also, most of the reported works have not highlighted the security vulnerabilities, attacks, with their respective requirements and solutions. All these reasons motivate us to give the following contributions in this research paper. This work is a good start, we will have all the novelties in the field of IoT, we will have the possibility of choosing the right path.

### Contribution

The objectives and originality/value of this paper are summarized as follows:

- The first research question is as follows: “which is the best AI technique or algorithm for improving IoT security?” for that, this paper performs a comprehensive study on the performance of AI algorithms regarding their accuracy;
- We studied previous works of AI algorithms, specifically the algorithms used in IoT security in order to perform an evaluation performance by comparing the accuracy of the results obtained in AI algorithms;
- This research paper gives a literature review related to AI, ML, DL, IDS and IoT security; it presents the state of the art of IoT attack classification and detection techniques based on AI;
- The main contribution is to choose an approach to secure IoT systems using the ML and DL after making a performance comparison between the different AI algorithms used in IoT security;
- This survey gives exploratory research on the AI and IoT security system. It also provides a comparative study of a recent IoT oriented datasets;
- The second question that can be asked is: “can we use the chosen technique to classify and/or detect intrusions and attacks in the IoT security systems?”. in the other hand, I precise the task (classification and/or detection) in which we can apply the chosen algorithm in IoT systems; it also efforts to show the performance study of AI algorithms-based IDS (Intrusion Detection System);
- It provides a comprehensive survey with a new and general taxonomy of supervised AI techniques. In this axis, it performs a new taxonomy of supervised classification methods used for IoT attack classification in order to give a general and a clear vision of the classification methods.

The value of this research in comparison to the existing studies is to be able to deduce or to know the novelty of each paper. The questions of research are: which is the best AI technique used for improving IoT security systems? which datasets are best for IoT security systems? For this reason, this paper provides a performance evaluation study and compares the many AI models that have been mentioned in the literature. This paper aims at getting some inspiring results and rational outcomes as well as to figure out which AI technique is best for classifying or/and detecting IoT attacks and intrusions.

### Outline

The remainder of this article is arranged as follows: The second section deals with the AI and IoT security. The third section presents the current state of the art. The fourth section presents the research methodology that covers a comparison in term of accuracy between the various AI algorithms used for IoT security systems. Afterward, the fifth section provides results discussion. Finally, the last section closes the article.

## AI and IoT security

This section is reserved to carry out a current state of our research subject based on several axes to help readers understand the contribution of AI to IoT security. To get a clear vision and provide a comprehensive survey in understanding the AI in IoT security, this section gives an overview on AI and IoT security taking into consideration several important keywords related to AI and IoT security including Machine Learning (ML), Deep Learning (DL), Intrusion Detection System (IDS), Classification, AI accuracy, and Prediction (best prediction accuracy). The following subsections focus on AI techniques in IoT security. This section can be helpful for the reader to get an idea about security attacks, vulnerabilities, requirements and solutions for each IoT layer and an overview of AI algorithms and its applications in the IoT security domain.

### Internet of Things (IoT) system

IoT is a vast domain which contains physical objects, networks communication, technologies, hardware (devices, computers), protocols, electronics, platforms and applications that interconnects anything from the physical environment (physical objects, animals, places, plants, machines and peoples …) to the internet in order to exchange data without any human interaction^3^. IoT covers many fields starting from human activities to industry eras. The main goals of IoT^3^ are to:

- Provide the best services for the human being, to allow many smartly new applications in the medical, industrial, economic, educational, and even individual daily life levels;
- Make human life more comfort and convenient;
- Save cost and time;
- Automate the interaction between environment and humans easily.

### Architecture and functioning of IoT system

The architecture of IoT system deals with multi-layer. There have been different proposals for IoT layers, each of which uses diverse technologies and bring a number of possible security issues. This subsection presents in detail the architecture of IoT that we proposed recently in^4^ to analyze security issues and problems. Moreover, we turn to the analysis of IoT attacks based on a four-layer architecture.

There is a lack of standardization in IoT architecture^1,3,4^. There are different proposals of the architecture of the IoT system. Some of these architectures of IoT system are composed of five layers^1,3^, while other architectures are composing of four layers^4^. For instance, the Cisco IoT reference model is composed of seven layers namely Collaboration & Processes, Application, Data Abstraction, Data Accumulation, Edge Computing, Connectivity, and Physical Devices & Controllers. The architecture of IoT that includes three layers namely physical/perception layer, network layer, and application layer would not be sufficient and not present in a great way the concept of the IoT, because it lacks cloud or middleware layer. Therefore, we need cloud or middleware layer, because we have a lot of continuous, enormous and sensitive data gathered from the Physical layer. Therefore, we recommend using the four-layer architecture. The underlying IoT architecture is composed of four layers, physical or perception layer; network layer; cloud/middleware layer; and application layer. In this research, we adopt the four-layer architecture because there are many advantages of using it:

- It is a thorough architecture;
- It reflects the IoT architecture;
- It is very general^1,3^ and presents in a great way the concept of the IoT^4^;
- Security at the middleware layer (storage/cloud/data) is not the same as security at the application layer (authentication/identification)^4^; as more than we separate the problems, we find solutions.
- The middleware layer is a necessary and an integral part of the IoT^4^. We need cloud because we have so much data generated by many connected objects.

**Figure 1:**
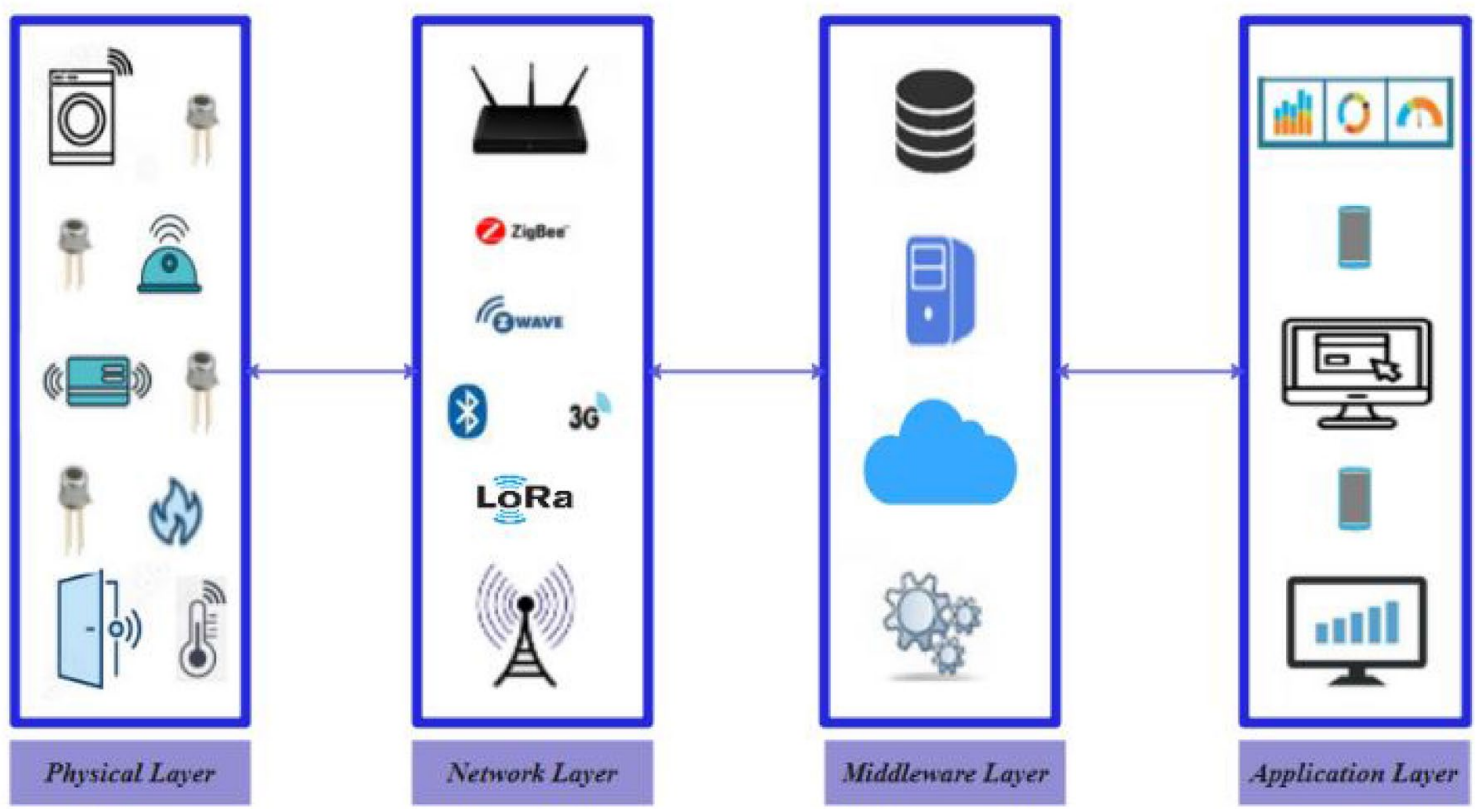
Proposed layers in IoT system^4^.

However, there is no standardized IoT architecture, and there is an inexistence of a secure IoT architecture. Figure 1 shows the four-layer architecture of IoT system and its corresponding technologies.

#### Physical layer

The main challenge of the perception/physical layer is the constrained devices on IoT systems^4^. This layer deals with sensors and devices which are used to receive/transmit data using different communication protocols such as Bluetooth, Zigbee, RFID, etc. RFID technology is mostly used for the automated information exchange between tags and readers using radio waves in the physical layer, it uses the Automatic Identification and Data Capture (AIDC) technology, attackers can destroy communication between RFID reader and tag, e.g., through RF jamming: RFID tags can also be compromised by kind of a DoS attack in which communication through RF signals is disrupted with an excess of noise signals. An attacker could physically modify the users of IoT system in order to get their sensitive data. Therefore, the exchanged sensitive data should be safe over heterogeneous networks and through constrained devices. The major security attacks that are encountered at the physical layer are as follows:

- *Eavesdropping*: Attackers can easily eavesdrop/sniff the device of the physical layer;
- *False Data Injection Attack*: The attacker captures the connected objects to inject erroneous data into the IoT system;
- *Unauthorized Access to the Tags*: tags can be accessed by someone without authorization. The attacker cannot just read the data but the data can be modified or even deleted as well, Due to the lack of proper authentication mechanism in a large number of RFID systems, however, RFID technology is vulnerable to many attacks such us Replay, spoofing, Tracking, Virus, Eavesdropping, Unauthorized access, Man in the middle, Killing Tag, Counterfeiting.

#### Network layer

Several communication networks and protocols are used for connectivity such as LoRa, WiFi, etc. Network attacks are multiple, usually affects work coordination as well as information sharing between devices. Concerning the network layer attacks, the sybil attacks^1,3^ are aimed at creating illusions in the network. At the network layer/level, the attacker targets the communication technologies of the IoT^4^ due to vulnerabilities of network protocols todestroy network communication. An attacker can send fake routing information to contaminate the entire network. The network layer is highly vulnerable to Man-in-the-Middle (MITM) attack. Blackmailer endeavors to mess up the network by launching the DoS attack. The impact of a DDoS attack^1^ (Distributed Denial of Service) is to disable the network. Moreover, the effect of security failures may contribute to the disruption of the whole network. Besides, the attacker can also exploit the lack of abnormalities and intrusions detection on networks. For that, an IDS should be implemented on this layer to monitor a network for malicous activities, as well as avoiding abnormal behavior of network participants and DoS attack that could lead to blocking the functionalities of part of the network. Furthermore, man-in-the-middle (MITM) attack can be caused by malicious node injection into the network. Therefore, an improvement of network intrusion detection using Artificial Intelligence (AI) is necessary. Besides, a new intrusion detection system (IDS) using AI techniques should be defined, implemented and validated on an actual case. Therefore, security on IoT networks is an important factor to consider. The main attacks against the network layer are the following^1,3,4^:

- *Phishing Site Attack*: The network layer for the IoT is very vulnerable to such attacks (phishing sites attacks). Phishing site attacks refer to attacks where multiple IoT devices are targeted with minimal effort from the attacker. An attacker pretends to obtain sensitive information such as the credit card details and passwords of users;
- *Selective forward attack*: the main goal of this attack is forwarding chosen packets by attacker to disturb routing paths;
- *Sinkhole attack*: In this attack, a malicious node may announce beneficial route or falsified path to attract so many nodes to redirect their packets;
- *Sybil attack*: In this type of attack, a malicious object may use different identities in the same network;
- *Wormhole attack*: This attack can be launched by creating private channel between two attackers in the network and forwarding the selective packets through it;
- *Blackhole attack*: a blackhole attack has been designed to drop silently all data packets that are meant to it by maliciously advertising itself as the shortest path to the destination during the path-discovering mechanism.
- *Hello flooding attack*: Objects recently joining the net-work send broadcast packet known as a hello message. In this case, an attacker can represent himself as a neighbor object to several objects by broadcasting hello message with a high-powered antenna to de
- *Denial of Service (DoS) attack*: is the most common attack that can affect network service availability and exhaust network resources to make it unavailable to users. It is a particular attack on a computational resource or on a network.

#### Middleware layer

Cloud is intended more for data analysis. We eliminate all the challenges of the first two layers (the perception layer and the network layer). This layer is about a distributed infrastructure to process and analyze IoT data^4^. It is also about decision making after eliminating and removing anomalous point from the data^3^ or anomalious group of data that may lead to false decisions, which help to reduce true negative rate and false positive rate. The major security attacks that are encountered at the Middleware layer are as follows:

- *Man-in-the-Middle (MITM) Attack*: Message Queuing Telemetry Transport (MQTT) is the most popular protocols utilized in IoT systems that transfer messages between two devices. It is an efficient and lightweight protocol that uses publish-subscribe communication model between subscribers and clients using the MQTT broker. Attacker can control the broker and become a MITM to gain complete all communications control without any knowledge of clients;
- *Signature Wrapping Attack*: XML signatures are used in the web services. The attacker modifies/falsifies the eavesdropped messages by breaking the signature algorithm and exploiting vulnerabilities in Simple Object Access Protocol (SOAP). The SOAP interface is offered by EC2 (Elastic Cloud Computing) to control deployed machines;
- *SQL Injection Attack*: The attacker uses SQL statements in the input data to delete, read, and write operations. Attackers can threaten the whole database system and obtain private data of users

#### Application layer

IoT computing provides the information that are visualized at this layer^3,4^. This layer is used to provide graphs for the decision and straightforward graphs on where the anomalies are? I.e. do we have anomalies in security? In the physical layer? As well as the control of the equipment, i.e., functioning or not functioning of the equipment, is the sensor which detects the temperature/humidity working or not? This layer process data from the cloud/middleware layer, as well as providing quality service to users. The major security attacks that are encountered at the application layer are as follows:

- *Buffer overflow* is one of the most used attack in different applications and software. The buffer overflow attack entails violation of the data buffer or bounds of code by exploiting program vulnerabilities.
- *Malicious Code Injection Attacks*: The attackers opt for the easiest method that they can use in order to break into a network. The attackers use Cross-Site Scripting (XSS) to inject malicious script into a trusted website. The XSS attack can paralyze the IoT system.

### IoT attacks classification

#### Security requirements and attacks classification based on IoT layer

Security issues in IoT systems, like restricted resources (or resources limitation in IoT devices)^1^, weak security design^4^, access control, configuration errors of the system^1^, compatibility problem^4^, heterogeneity^4^ (due to the different components of IoT environment with different characteristics and different communication technologies), scalability^3^, Big Data problem^4^, poor updates, the generation of continuous and enormous sensitive data over time (every second/minute/hour/day/week)^4^, interoperability^3^, inexistence standardization and lack of secure IoT architecture^1^, are the main challenges. To secure IoT environments, Lightweight Cryptography, Access Control, Encryption, Key Management, Intrusion Detection System (IDS) are some security solutions for IoT. Further, confidentiality, integrity, availability, privacy, authentication, authorization, non-repudiation, authenticity, identity, and compatibility are the main IoT security requirements^4^. We adopt in this research the four-layer architecture^4^ (physical, network, cloud or middleware and application layer) to classify attacks. More in detail, these requirements are discussed in^4^. We propose a layer-based classification of vulnerabilities and threats on IoT. Therefore, a proposal for a secure IoT architecture (the most secure protocols…) is needed.

**Figure 2:**
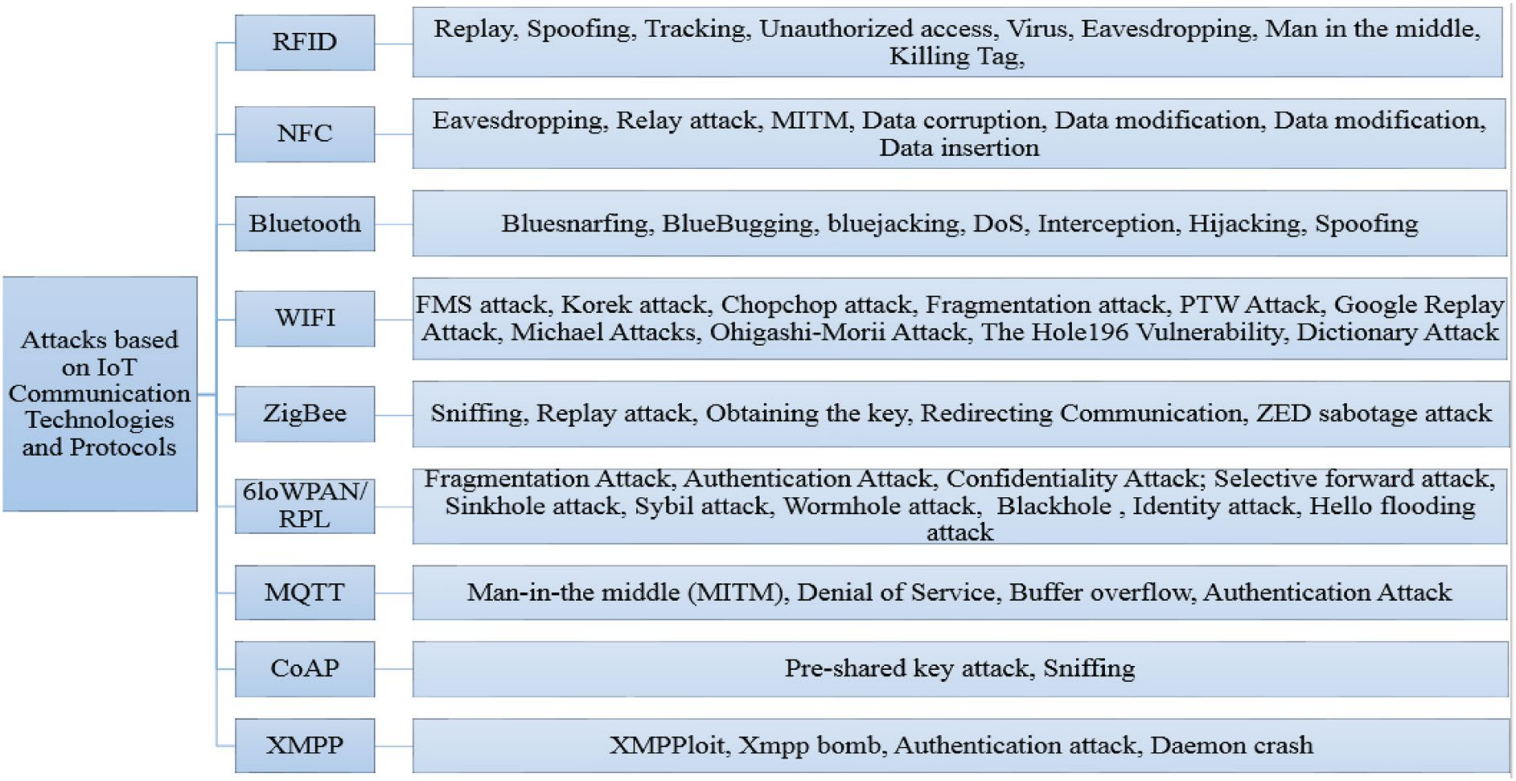
Taxonomy of IoT communication technologies and protocols attacks.

Table 1 represents security requirements, solutions and IoT attacks based on layer classification. It explores the vulnerabilities, threats and attacks in the IoT environment and the security solutions that can be implemented in the IoT layers. It aims at classifying the potential IoT threat and vulnerabilities by an architectural view in order to get a clear vision on how to address security requirements for each layer to avoid the current IoT threats on each layer. i.e., in the IoT environment we classified the security solutions, requirements for each layer separately. Indeed, the study of vulnerabilities on the layers of IoT systems was discussed in^4^. While, Fig. 2 shows classification of attacks on different communication technologies and protocols. More, the study of communication protocols of IoT systems were discussed in^1,3,4^. From the Table 1, we conclude that the CIA (Confidentiality, Integrity, Availability) presents the most critical services to consider when implementing a solution to secure the IoT system. Therefore, we need to have a clear idea about the security solutions that we need to implement in a specific IoT layer in order to improve IoT architecture security. i.e., we need to secure the layers in order to secure IoT as whole. Thus, several attacks, i.e., Eavesdropping, Physical damage, Man-in-the-middle (MITM), are the most attacks in the physical layer. To overcome these issues, attack detection could be deployed.

**Table 1:** Security requirements, solutions and Attacks classification based on IoT layer.

Layer	Physical	Network	Middleware	Application
Attacks, threats and vulnerabilities in IoT^1,3,4^	Eavesdropping, Spoofing attack, Man in the middle, Tag cloning, Node Capture, Cryptanalysis attack, Social engineering, DoS (Denial of Service) Attack, Denial of Sleep Attack, Distributed DoS Attack, Fake Node, Replay Attack, Routing Threats, Side-Channel Attack, Mass Node Authentication, Jamming of Node in WSN, Man-in-the-middle (MITM), False Data Injection, Unauthorized Access to the Tags etc	Sleep deprivation attack, Data Transit Attacks, Cryptanalysis attacks, Sybil attack, sinkhole attack, wormhole attack, Hello flood attack, Spoofing attack, DDoS Attack, Altered, spoofed or replayed routing information, Phishing Site Attack, Selective forwarding, Blackhole attack, DoS, etc	Shared resources, DoS, third-party relationships, underlying infrastructure security, MITM, application security, virtualization threats, Signature Wrapping Attack, SQL Injection, etc	DoS, Data protection and recovery, Buffer Overflow, malicious scripts, virus, worms, spyware injection, Unauthorized Access, malicious code injection attacks
Security requirements in IoT^1,4^	Confidentiality Integrity Availability Authentication Authorization	Confidentiality Integrity Availability Authentication Authorization Non-repudiation Privacy Compatibility	Confidentiality Integrity Authenticity	Integrity Authentication Authorization Privacy Identity
Security solutions^3^	Lightweight cryptography, Key Management	Intrusion Detection System, Communication protection, Key Management	Secure Cloud, Access Control	Access Control

The classification of security attacks within IoT is designed to help IoT developers raise awareness of the risks of security threats and flaws so that better security solutions shall be incorporated. The most common security solutions in the physical layer of the IoT architecture includes Lightweight cryptography, Key Management (Table 1). In Table 1, we give the proposed solutions and requirements which extracted from the recent researches^1,3,4^ for each layer of the IoT architecture.

#### Attacks classification based on IoT communication technologies and protocols

As discussed in Refs.^1,3^, different communication technologies can be used to exchange and transfer data. IoT systems are heterogeneous, it uses different technologies which are not limited to, IPv6, Zigbee, 6LoWPAN (IPv6 Low power Wireless Personal Area Network), Bluetooth, NFC (Near Field Communication), Z-Wave, RFID (Radio Frequency Identification). However, these technologies are susceptible to several attacks.

The different communication technologies in the IoT systems allows objects to interconnect with each other in order to exchange data (capture, process, store, transfer data between the elements of the first layer of the IoT architecture and the other layers)^1^. Communication technologies can be categorized according to^1,3^:

1. “Short Range Communication Technologies” including: RFID, NFC, WSN, 6LowPan, Z-Wave, Zigbee, Bluetooth, Wi-Fi, BLE;
2. “Long Range Communications Technologies” or “LPWAN” including: Sigfox, LoRa/LoRaWAN, NB-IoT;
3. Cellular communication: 2G, 3G, 4G, 5G.

These technologies can be divided into three groups: Short Range, Long Range or LPWAN (Low-power wide area network) and Cellular communication. Each of these technologies in an IoT system bring a number of security attacks. Figure 2 shows various possible attacks on these technologies according to^5^.

As mentioned before, MQTT is the most widely utilized telemetry protocol in IoT^1,3,4^. If this protocol is not secure, it cannot be used like that. Therefore, an analysis of the protocol concerning (analysis of sending data, receiving data) is necessary. Hence, if the protocol is not secure, then, it must be encapsulated with a security layer SSL^1^.

### Artificial intelligence (AI)

AI is about choosing the right decision at the right time. The domain of application of AI has become unlimited. Therefore, any field that exist now benefits from AI. AI in IoT Security? AI is a leading technology, which studies ways to build intelligent programs and machines that can creatively solve problems, it can be used to improve accuracy rate and prediction results. AI domains can include ML, NLP (Natural Language Processing), Robotics, Expert systems, Vision, Speech, etc. AI can also be used for IoT security and intrusion detection. In our study, we are interested in the use of AI (ML and DL) for IoT security. Therefore, the proposal of an IDS based on the chosen algorithm(s), then, the development and improvement of one or more reliable algorithms are needed, and finally, the integration of the IDS for adaptation to IoT systems is needed. Moreover, AI algorithms can also be exploited to create an anomaly detection system in IoT. Other than intrusion detection, the AI methods studied in this paper have been successfully applied in other areas, such as:

- Healthcare: AI becomes a key factor in healthcare field, especially in surgical assistance, early diagnosis and prevention.
- Industry: many AI use cases can be cited like robotics, and autonomous driving in automotive.
- Education: Intelligent teaching and tutoring as well as science simulation are some noted example of AI application in education.
- Digital Marketing: in order to provide high data quality, AI can be used for analyzing the collected data using different techniques like ML and classification.
- NLP (Natural Language Processing), speech recognition, image processing, are other research fields.

AI has become a topic of the hour and extends more since its continuous development and performance in classifying and detecting IoT attacks. Further, it becomes more relevant for improving IoT security systems. For example, there are many AI techniques that can be used for an intrusion detection system (IDS). i.e., AI algorithms can be exploited in building/developing intelligent security mechanisms, they can be used in anomaly detection, intrusion detection, etc. Furthermore, AI can be used as plugins into open source IDSs to improve network intrusion detection. Concerning AI types, it has different forms: strong AI, narrow AI, and hybrid AI.

- Strong AI: also called the full AI or Artificial General Intelligence (AGI). A Strong AI is a type of AI that replaces human intelligence. This type of AI has not yet been achieved and is not addressed for a specific issue;
- Narrow AI: also called the weak AI which is designed for a specific problem. This type of AI lacks the human intelligence flexibility;
- Hybrid AI: AI-based solutions that combine multiple weak AI in order to try to create a strong AI.

**Figure 3:**
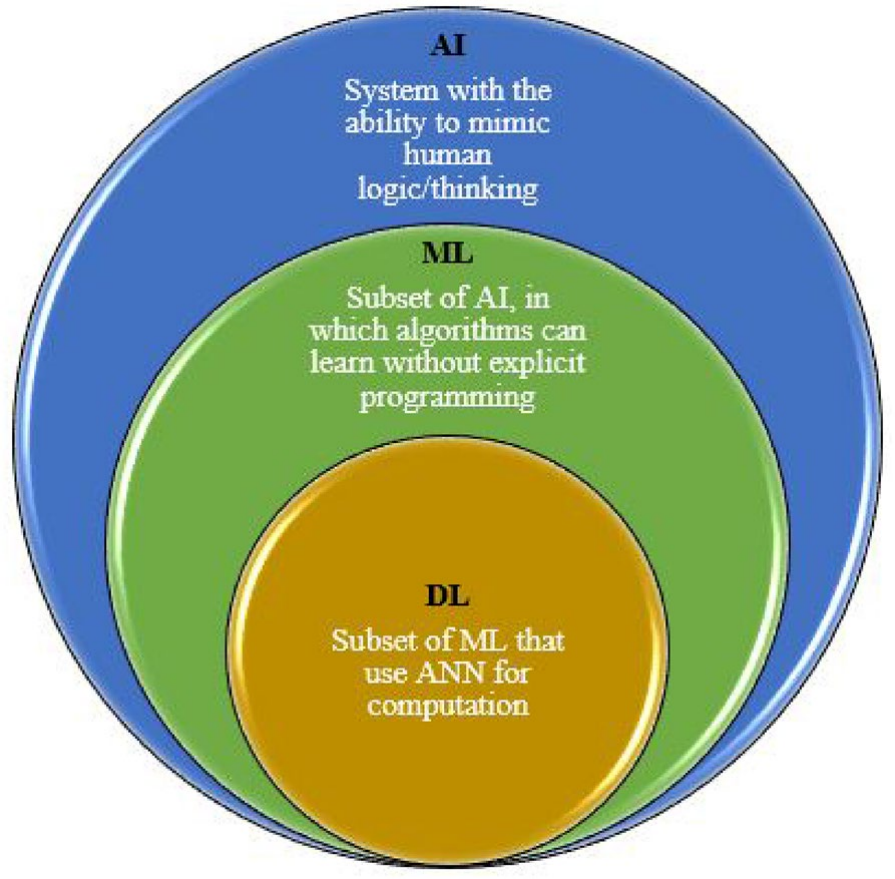
AI/ML/DL difference.

AI is the science of making machines do things that requires human intelligence. It is a system that attempts to mimic human thinking. AI raises basic issues about information processing, human intelligence, the mind/body problem, memory, symbolic reasoning, the origins of language, etc.^6^. AI plays a significant role and it is a good tool to adopt for securing IoT systems. AI is being used for improving IoT security systems as well. For example, several studies used AI techniques to detect attacks and develop an efficient IDS. Indeed, the most recent findings assume that these AI algorithms can be exploited for the creation of an anomaly detection system in the IoT. Therefore, AI is a tool or key solution based on various ML techniques. DL is a subset of ML based on ANN (Artificial Neural Network). The difference between the ML and DL is as follows: to complete the task, ML requires some direction, while DL does not require the intervention of any programmer. Moreover, the software must control the ML, while, DL might learn on its own. The bellow Fig. 3 shows the difference between AI, ML and DL. This following two subsections will discuss ML and DL techniques for IoT security.

#### Machine learning (ML)

**Figure 4:**
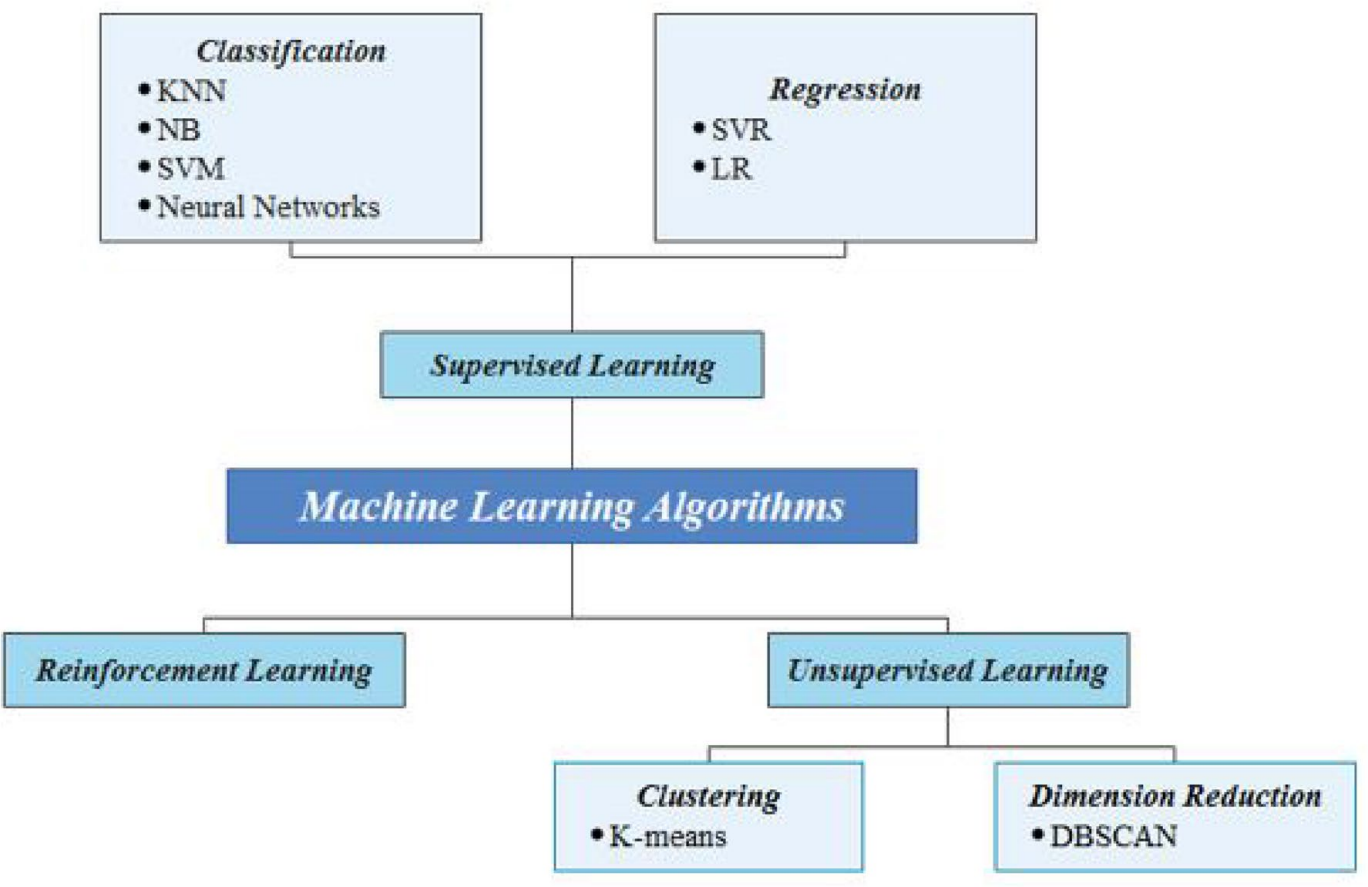
Taxonomy of machine learning algorithms used for the security of IoT systems.

ML is a subset of AI, in which algorithms can learn without explicit programming. Therefore, ML techniques has been widely used to handle “Big Data”. Additionally, ML assumes a fundamental role in the IoT aspect to manage the immense volume of information/data produced by IoT objects. ML approaches like (k-Nearest Neighbors (KNN), Decision tree (DT), Naive Bayes (NB), Support Vector Machines (SVM), Random Forest (RF), Logistic Regression (LR), Support Vector Regression (SVR), etc.) could be included supervised, unsupervised, and reinforcement learning. The number of layers in a neural network might vary depending on how difficult the problem to be solved is. There are several types of the neural networks including Multi-Layer Perceptron (MLP), Artificial Neural Network (ANN), Recurrent Neural Networks (RNN), Convolutional Neural Networks (CNN). The neural networks can be used for improving the classification of attacks in IoT domain. Since, this different types of it were exploited in several scientific research works as a classification and detection of potential attacks in IoT system. Figure 4 shows a taxonomy of machine learning algorithms used for the security of IoT systems. ML algorithms can be divided into three major categories:

- Supervised algorithms: in which the training set contains labeled data, so the goal of the algorithm is to make it once trained able to give its predictions on unlabeled data. In other words, the goal of the algorithm is to give its predictions on unlabeled data by using the training set. There are two types of this class including classification and regression. Supervised learning algorithms includes DT, K-NN, SVM, Naïve Bayes, NNs, etc.
- Unsupervised algorithms: The data are unlabeled, and the algorithm must be able to find out the similarities between this data. K-means and DBSCAN (Density-Based Spatial Clustering of Applications with Noise) are examples of unsupervised algorithm.
- Reinforcement algorithms: allows the machine to learn by interacting with its environment. Q-learning is an example of reinforcement learning. Reinforcement Learning techniques have been used to secure IoT devices by detecting various IoT attacks.

#### Deep learning (DL)

**Figure 5:**
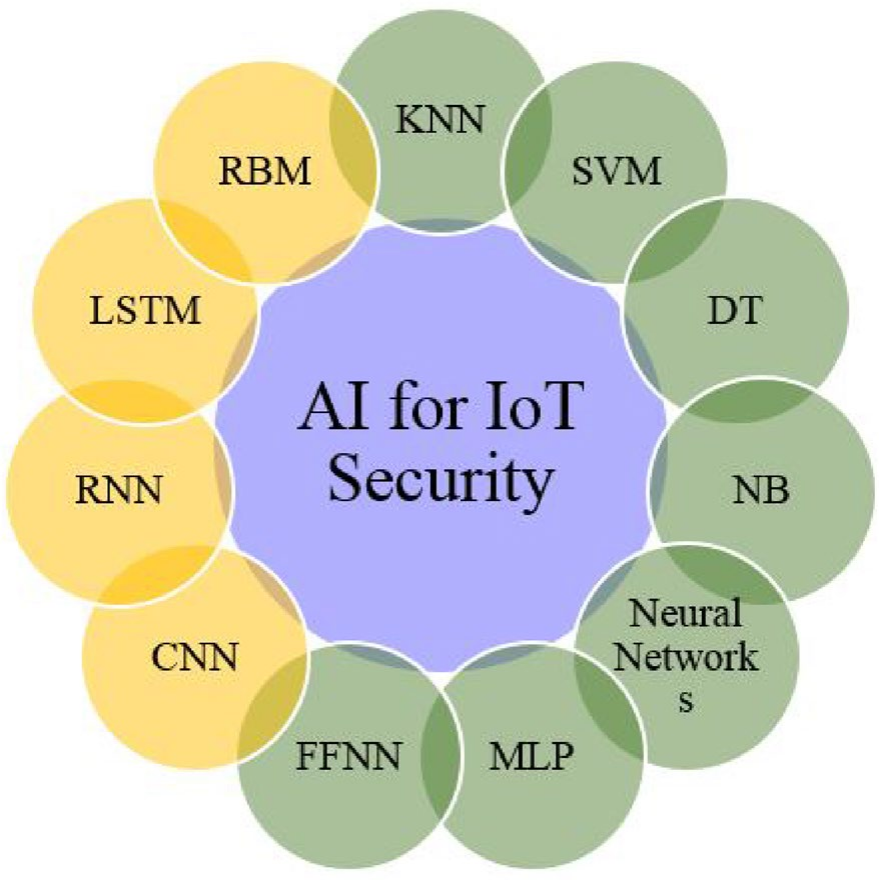
AI approaches for IoT security. *KNN* k-Nearest Neighbors, *SVM* Support Vector Machines, *DT* Decision tree, *NB* Naive Bayes, *MLP* multilayer perceptron, *FFNN* Feed forward neural network, *CNN* Convolutional neural network, *RNN* Recurrent neural network, *LSTM* long short-term memory, *RBM* Restricted Boltzmann Machine.

DL is a subset of ML that use ANN (Artificial Neural Network) for computation. DL is composed of deeply layered neural networks. The term “deep” refers to the number of hidden layers in the NN, that is why DL models are often referred to as DNN. DL models can be categorized into supervised, unsupervised, semi-supervised. Feed Forward Neural Network (FFNN), Convolutional Neural Networks (CNN), Recurrent Neural Networks (RNN), Long Short-Term Memory (LSTM), Gated Recurrent Unit (GRU), Generative Adversarial Networks (GAN) are some of the best-known DL algorithms. The CNN is composed of input, convolution, pooling, and fully connected layer. The LSTM method works in three stages: Forget Gate, Update Gate/input gate, and Output Gate. The particular AI algorithms used in security is also discussed. Figure 5 shows the AI approaches for IoT security. The two subsets of AI are ML and DL. These parts of AI are discussed and detailed in the fifth section. This survey deals with AI methods in IoT security systems. For example, HIDS (Host Intrusion Detection System), NIDS (Network Intrusion Detection System), and anomaly detection are common IoT security application fields where DL has been applied prominently. Actually, DL has taken a place in the fields of cyber security and IDS. This paper highlights the importance of these AI algorithms in providing security against attacks and intrusions in the IoT context. For that, this paper covered a state of the art on IDS (Intrusion Detection Systems), types, etc.

### Classification and detection methods

#### Classification with AI techniques

It is a process of categorizing a given set of data into classes^3^. Classification technique is a supervised learning technique, in which the data is labeled. In other words, when the classes are predetermined and the examples known, the system learns to classify according to a classification model. These classes can be multitudinous or binary (such as anomaly detection). Classification assigns a category/class to data whose class is unknown. Classification problems consist in classifying attacks according to classes. There are various algorithms of classification including: KNN, SVM, NB (Naive Bayes), NNs, etc. Classification algorithms can be applied to detetct several types of IoT attacks to enhance and improve the performance of IoT IDS. Classification plays a significant role in building a performant IoT IDS to differentiate between the different types of IoT attacks. For this, the performance of these classification techniques must be tested. Moreover, classification is a supervised Machine Learning technique, which helps to secure the system and detect real attacks. It aims to make a prediction whether the data is properly classified or not. To do this, a training/learning set is used to train a classifier (classification algorithm), this learning set will also be applied to a test set containing similar data to classify and make prediction. Thus, based on the classification, intrusions can be detected. i.e. classification is used for IDSs based on multiple or binary classes. Therefore, choosing the best classification algorithm (AI algorithm) is based on performance evaluation/assessment in terms of accuracy rate. The primary challenge of classifying/identifying the correct categories/classes is finding the correct ratio/rate between the training and test sets.

#### Intrusion detection system (IDS)

Is a mechanism intended to detect abnormal or suspicious activities on the analyzed target (a network or a host). It thus makes it possible to have a preventive action on the risks of intrusion. In other words, it is software, hardware or a combination of both that aims to monitor a single host or network for malicious activities and to track and supervise the network security against any policy violation and cyber attacks. For instance, securing an IoT architecture by installing IDSs at its layers is a new solution to tackle security threats and vulnerabilities. Indeed, to enhance IoT security, the integration of IDSs into IoT system layers is required. In addition, IDS can be utilized in a healthcare use case in order to monitor human health and control human body by implementing sensors in blood, heart (WBAN Wireless Body Area Network). Therefore, an improvement of the IDS is required for adaptation to IoT systems. More, IDS is widely used to detect abnormalities and intrusions on networks. Basically, sensors can be implemented in clothes, heart of human to control its health condition. For example, Tesla has proposed a chip implemented in brain to control the health status of a human, i.e., Elon Musk hopes to be able, through his company Neurolink, to implant chips in the human brain from 2022 which is intended for medical use. Therefore, to test the operation of this chip, this system must include an intrusion detection system or an anomaly detection system or an analysis system. However, complex adaptive AI systems conduct the risk of promoting self-sustaining evolution of malicious systems that can imitate a cancerous development in the human body.

**Figure 6:**
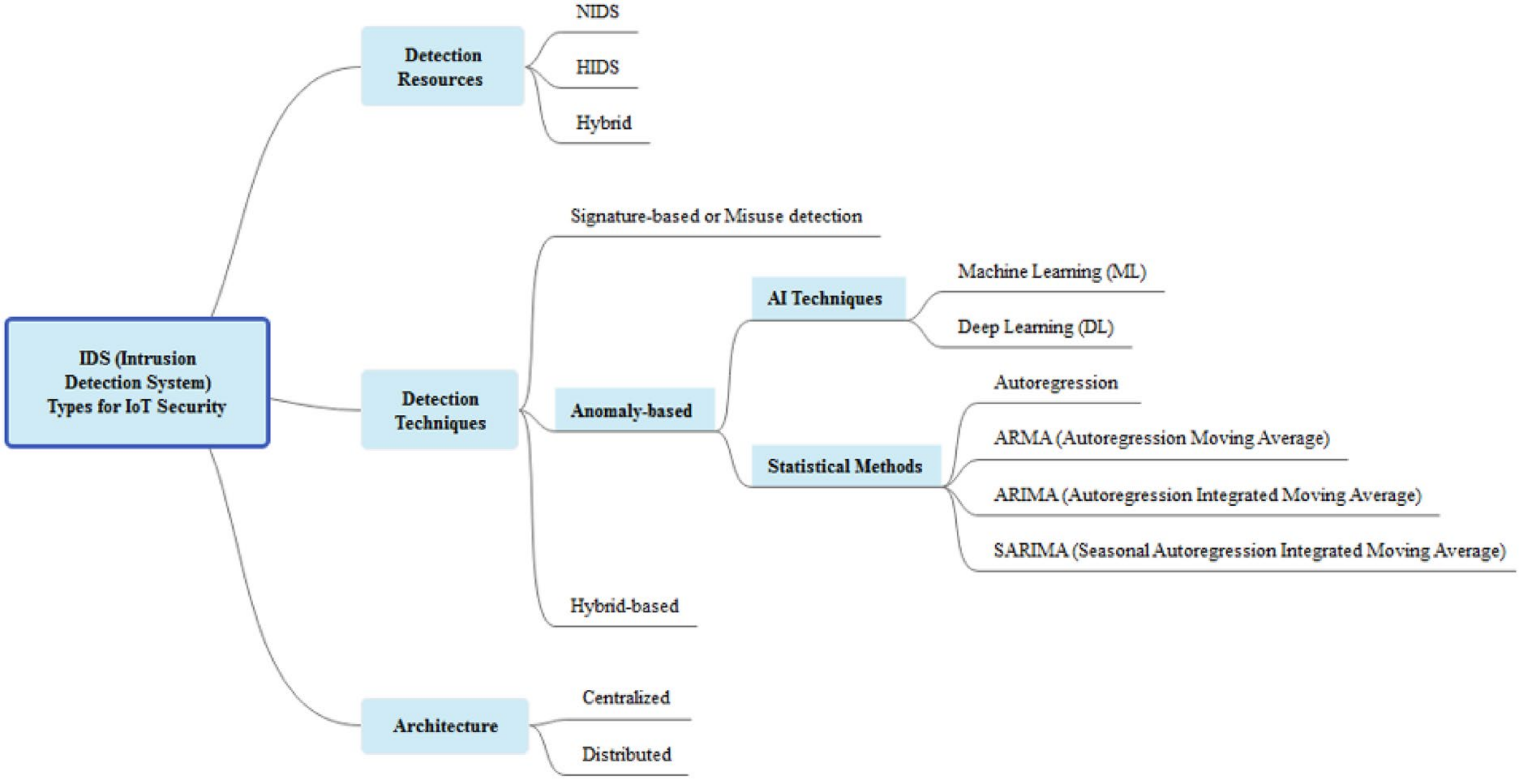
Intrusion detection system types for IoT security.

Detection is a kind of classification with two neurons in output (Normal and Attack). To detect intrusion, AI techniques must be able to classify abnormal and normal network behaviors. To get the best results, many AI techniques can be used to do intrusion detection in IoT system. Its main objective is to protect users from such attacks using AI techniques. Detecting attacks can be done by using classification. For instance, if we take an input, firstly, we detect whether it is normal or attack, secondly, we identify the class of the detected attack. Intrusion detection system (IDS) is a prevention mechanism on the risks of intrusion intended to detect abnormal activities on a network or a host. False positives and False negatives rates are the two fundamental concepts, the first one means an alert coming from an IDS but which does not correspond to a real attack (i.e., normal traffic is considered an attack.), the second is about a real intrusion that went undetected. The outlier detection or anomaly detection helps to minimize the true negative and false positive rates, which means it helps reduce AI false predictions and improve results. It also plays a significant role in securing IoT systems or any security system. Hence, IDSs are extensively used to keep track of harmful activity on a network or on a single computer. Its aim is to identify or find vulnerabilities and notify the system administrator or gives immediate alerts of any potential threats or attacks. Machine Learning and Deep Learning techniques can be used for detecting attacks for IoT. A comparison between the different types is needed. Figure 6. shows intrusion detection system (IDS) types for IoT security. IDS based detection resources can be arranged into three categories:

- Network based (NIDS-Network Intrusion Detection System): a NIDS listens to all network traffic, then analyzes it and generates alerts if any packets seem dangerous, it aims at detecting intrusions in real time. NIDSs are used to protect a company's IT assets.
- Host based (HIDS-Host Intrusion Detection System): a HIDS is based on a single machine, this time no longer analyzing network traffic but the activity happening on this machine. It analyzes in real time the flows relating to a machine as well as the logs.
- “Hybrid” intrusion detection systems: they are able to gather much more information from a HIDS system than a NIDS. Typically used in a decentralized environment.

For detection methods in IDS, it can be arranged into three categories:

- Signature-based methods: contains a database of known attacks signatures which can be identified by this approach. However, this method cannot detect a new attack;
- Anomaly-based intrusion detection system: A system error can lead to abnormal behavior. The anomaly-based detection method aims to define the behavior of data and create a model as a knowledge to compare real records with abnormal behavior. This technique can detect new attacks and threats; it does not need database updates. We conclude that anomaly-based IDS is the best method for detecting new attacks unlike signature-based IDS and rule-based IDS despite the fact that it gives high true negatives and false positives rates. For example, anomaly detection can be applied for verifying the accuracy/integrity of the transferred data in the network layer which improves the security of the physical layer. Moreover, data integrity should be ensured. Algorithms for anomaly detection can be statistical methods, AI-based methods, etc.
- Hybrid-based intrusion detection system: it combines both signature and anomaly-based intrusion detection system to detect new intrusions.

### AI accuracy

Accuracy is an evaluation metric which is used to test models. It represents the overall effectiveness of the classification model. It is the ability to appropriately distinguish between intrusions and normal behavior. This work aims at making a comparison between the accuracy rate of different AI algorithms. The evaluation step aims at estimating the performance of the model on a test dataset (new input/data). The accuracy depends to the data preprocessing as well as the quality and to the quantity of data. The performance of the AI techniques has been evaluated based on accuracy metric, because all the selected papers use the “Accuracy” metric to evaluate their AI models. In terms of evaluation metric of AI models, we notice that the “accuracy” was the major metric and the main factor used than “precision”, “recall”, “F1 score”, “Loss”, and “AUC (Area Under the Curve)”. More, the evaluation of accuracy requires the use of an IoT-related dataset to reflect realistic/real-world IoT applications. Further, before applying any AI method, we need to clean and prepare the data in order to accelerate the learning process and achieve good AI accuracy. In order to prove that this is the best/right AI method to do IoT attacks classification and detection, we perform the performance assessment of multiple AI methods in IoT security.

## State of the art

This section will review some researches on AI techniques used for IoT security to give more idea about the current state of the art on AI techniques and methods used for IoT attacks classification and detection. To overcome the security problem of IoT systems, and in particular, the classification and detection of IoT attacks and intrusions, many AI techniques have been proposed by several researchers. This literature review focuses on AI-based security for IoT systems until the most recent papers published in this area till 2023. This section consists in presenting the state of the art of IoT attack classification and detection techniques as well as detecting intrusions in an IoT system using AI. In this contribution, the objective is to be able to outline the methods used for classification or/and detection of attacks in IoT environment using AI and related in particular to ML/DL or other techniques such as the CTM method, steganography.

In 2021, Meziane et al.,^1^ and^3^ proposed a new classification based on Classification Tree Method (CTM) to classify IoT attacks. It makes the evaluation of IoT systems more systematic and allows selecting attack test cases through CTE (Classification Tree Editor) tool. They try to solve the problem of classification. They exploit the usability of Classification Tree Editor (CTE) editor to generate appropriate test cases. In their research they present two approaches to improving the IoT systems evaluation process:

- A systematic method to generate test cases;
- Selection of test cases based on appropriate attack classification.

Although this method has several advantages including, all possible test cases are identified and relevant test cases are selected in a systematic manner, that helps to eliminate/reduce some errors and makes easier its management. However, the CTM method has a number of drawbacks. i.e., Although the CTM method has various advantages, nevertheless it suffers from some drawbacks which are mentioned in fifth section. Additionally, the CTM method has not been classified by any author in the previous works. For this reason, I also suggest adding or classifying the CTM method in the fifth section. The proposed classification of IoT attacks will be helpful for detection.

In 2022^4^, Meziane et al., proposed an IoT architecture that includes four layers: the physical layer, the network layer, the middleware layer, and the application layer. The study covered the architecture of IoT and the different technologies used, including communication protocols such as LoRa, LoRaWAN, 5G, as well as the characteristics and specifications of each layer in an IoT system. At each layer, the attacks and vulnerabilities are described and illustrated in detail. Moreover, the defined IoT architecture and its security requirements are also discussed in detail. The research performs the comparative study on IoT architecture and well-defined attacks. The results shows that we need to focus on securing the physical and the network parts. Because generally it is the parts that contain the big challenges. The resource challenge in the first layer (Physical or Perception layer), and the challenge of data heterogeneity in the second layer (Network layer).

In 2019^7^, O. Ibitoye, O. Shafiq, and A. Matrawy, used two DL models: FFNN (Feed Forward Neural Networks) and SNN (Self-normalizing Neural Network) to classify the intrusion in IoT network. The authors compared also the detection performance between SNN and FFNN using BoT-IoT dataset. As a results, FNN outperforms SNN. FNN get better results in terms of precision, accuracy, and recall. However, authors evaluated their models on only a single dataset.

In 2018^8^, Y. Zhou, M. Han, L. Liu, J.S. He, Y. Wang, have proposed a model for cyberattack detection in the IoT environment. To predict the intrusions, they trained the deep FFNNs using the back-propagation algorithm. For the NSL-KDD dataset, the accuracy has been above 95%, but for the UNSW-NB15 all models have achieved an accuracy < (less than) 95%. However, the used dataset may not represent IoT network traffic. i.e., NSL-KDD does not provide satisfactory results. Because it has two main issues: (1) it lacks recent normal traffic patterns, and (2) it does not cover recent attack patterns.

In 2018^9^, T. Aldwairi, D. Perera, and M. A. Novotny, evaluated the suitability of the applied RBM (Restricted Boltzmann Machines), to distinguish between abnormal and normal NetFlow traffic. They evaluated their approach by testing it on the renowned ISCX (Information Security Center of Excellence) dataset. Their results showed that the proposed method can be trained successfully for classifying anomalous and normal NetFlow traffic. However, the model is not compared with other similar models.

In 2017^10^, K. Vimalkumar and N. Radhika, used various ML techniques to create a big data framework, while, intrusions are detected using classification methods (DNN, SVM, RF, DT, NB). Based on the classifications, the intrusions were detected on the synchrophasor dataset. Even thought, the DNN model gets the highest accuracy (79.86%) than the other techniques used in their work, but this accuracy is < (less than) 80%.

In 2020^11^, Alsaedi, A., Moustafa, N., Tari, Z., Mahmood, A., & Anwar, A., evaluated the performance of several popular ML methods and a DL model in both multi-class and binary classification for intrusion detection purposes by the proposed Telemetry dataset for Industrial IoT (IIoT) and Industry 4.0/IoT. Moreover, due to the lack of benchmark IIoT and IoT datasets for assessing IDSs-enabled IoT systems, they proposed and described dataset, which is called TON_IoT. Besides, for evaluating the performance of seven supervised Machine Learning methods, various evaluation metrics (i.e., recall, accuracy, F-score and precision) were used. CART achieves good results than other techniques with a score of 77% for all the metrics, as well as 75% for F-score. The main finding of this evaluation was that CART and RF achieved the highest score in all metrics. Compared to the other methods, the results have also shown both KNN and LSTM had the second-best performance.

In 2019^12^, Y. Zhang, P. Li, and X. Wang, present in their work an intrusion detection model in IoT based on GA (genetic algorithm) and DBN (deep belief network). Moreover, the NSL-KDD dataset was used to evaluate algorithms and the model. For detection, this method reaches more than 99%. However, the used dataset does not target IoT system, which means that the used dataset may not represent IoT network traffic.

In 2020^13^, Ferrag et al., analyzed seven DL models including RNN (recurrent neural networks), DNN (deep neural networks), RBM (restricted Boltzmann machines), DBN (deep belief networks), CNN (convolutional neural networks), DBM (deep Boltzmann machines), and DAE (deep autoencoders). In addition, the authors used DL models for intrusion detection. Furthermore, they studied the performance of models in binary and multiclass classification (two categories of classification) using two new real traffic datasets, namely, the Bot-IoT dataset and the CSE-CIC-IDS2018 dataset. They have used three important performance indicators, detection rate, accuracy, and false alarm rate.

In 2019^14^, Ferdowsi et al., proposed a distributed GAN (generative adversarial network)-based IDS solution to detect intrusion in the IoT. In terms of accuracy, they show the superiority of their model, which has up to 25% higher precision, 20% higher accuracy, and 60% lower false positive rate compared to a standalone GAN-based IDS.

In 2019^15^, Liang et al., studied the application of a NN (neural network) in IDSs (intrusion detection systems), they used NSL-KDD dataset to test the proposed system. They applied a DNN technique for intrusion detection. The DNN has a good performance (accuracy rate was 97%) for detecting intrusions in an IoT environment.

In 2019^16^, Ge et al., presented in their work a Deep learning-based intrusion detection for IoT networks. The authors used FNN an intelligent binary and multiclass classification, but with just a few classes to get 99% in all evaluation measures (accuracy, precision, recall, and F1 score) for DDoS/DoS attacks while the normal traffic classification got an accuracy of 98%.

In 2019^17^, according to Yuan et al., the precision can reach 96% in binary classification about anomaly and normal. AC-GAN (Auxiliary Classifier Generative Adversarial Network) is also adopted. Results show that their technique could improve the precision of network traffic classification. The classifier they used has a good performance on the classification between anomaly and normal. They obtained a recall of 98% in anomaly traffic detection. However, no comparative analysis of other similar works for the evaluation of the model.

In 2019^18^, Nagisetty and Gupta used four different DL models such as MLP (Multi-Layer Perceptron), CNN (Convolutional Neural Networks), DNN (Deep Neural Networks) and AE (Autoencoder). In IoT networks, the authors aim at detecting malicious activities. Their performance evaluation is done using two datasets: NSL-KDD99 and UNSW-NB15 and they discussed their result analysis in terms of accuracy.

Sainis et al.^19^ used five ML classification algorithm including NB, KNN, SVM, DT (C4.5) and RF for the attack classification problem under three datasets called NSL-KDD, KDD cup'99, and GureKDDcup. However, NSL-KDD and KDD Cup 99 do not provide satisfactory results. Because it has two main issues: (1) it lacks recent normal traffic patterns, and (2) it does not cover recent attack patterns. Moreover, authors used KDD Cup’99 dataset which is an outdated dataset.

In 2018^20^, Nazim Uddin Sheikh et al., discussed a signature-based IDS for IoT environments. The NSL-KDD dataset is used for testing their system. The proposed IDS consists of four components: Signature Generator, Pattern Generator, Intrusion Detection Engine, and Output Engine. In their experiments, they evaluate the false negative and false positive occurrences.

In 2018^21^, Diro et al., have adapted an LSTM to detect cyber-attacks. Their experiment was conducted on two datasets AWID (Aegean WiFi Intrusion Dataset) and ISCX datasets. The AWID dataset and the ISCX dataset both showed a similar trend for all measures (accuracy, recall, and precision). Furthermore, the precision-recall curve of LSTM is greater and higher than that of LR (logistic regression), which means that the system ideally and perfectly classifies normal and attack instances into their respective classes/categories. The obtained high values of recall and precision may be due to low false negatives and false positives, respectively. A low false positives and false negatives indicated high relevance in attack detection systems.

In 2020^22^, Kasongo et al., proposed a FFDNN (Feed-Forward Deep Neural Network) for binary and multiclass types of attacks, the efficiency of the model was tested using AWID and the UNSW-NB15 datasets. The proposed method was high compared to those algorithms k-Nearest Neighbor (kNN), Random Forest (RF), Naïve Bayes (NB), Decision Tree (DT), Support Vector Machine (SVM). Their experiment demonstrated that the proposed method obtained respectively 99.77% and 99.66% in accuracies for the multiclass classification and the binary. Their proposed approach has greater detection in accuracy than other techniques.

In 2019^23^, Hwang et al., used LSTM (Long Short-Term Memory) network model, their aim was to classify an incoming packet is a part of malicious traffic or normal. They have used the USTC-TFC2016, Mirai-RGU, ISCX2012 and Mirai-CCU datasets. They have assumed that the performance of theirs is competitive on classifying flows into malicious or benign with the prior work. In addition, authors have many interests in the classification of the incoming packet whether it is malicious or not, instead of considering the attack type in detail. The results show that the LSTM method reached 100% accuracy.

In 2019^24^, Ferrag et al., employs RNNs (recurrent neural networks) for detecting network attack. Three different sources the Power System dataset, a CICIDS2017 dataset, and a Bot-IoT dataset were studied for the performance of the proposed IDS. The better accuracy for CICDS2017 dataset is 99.811%.

In 2019^25^, Koroniotis et al., focused on AI-based detection algorithms for IoT networks. They proposed a new dataset called Bot-IoT, they carried out binary classification and their prediction output of models was classified as normal traffic or attack. They compared their dataset to other datasets that were publicly available. The proposed dataset was claimed to be the only one in the comparison that include IoT traces. To evaluate the quality of the new dataset, SVM, LSTM, RNN were utilized to train a classifier. The obtained results show that the Bot-IoT can be utilized to train accurate models, with RNN and LSTM outperforming the SVM implementation. However, they did not classify the output into the different categories of attacks.

However, each time an article appears which tests on a data (network data KDD Cup 99) and gives a new accuracy and precision, which is insufficient. Because, they tested on a data which does not reflect the reality of IoT. Recently, there appear new and specific datasets for the IoT, it is a need. To obtain the best performance results, we extracted the information from these papers^7–19,21–35^ as illustrated on Table 2. After that, we summarized, analyzed and compared these results found by focusing on the used dataset(s), the AI algorithm(s), and the accuracy. The main results of this research are discussed in the Section V.

**Table 2:** Recent works on AI in the IoT security.

Papers	Year	Objectives/Purpose	Dataset(s)	Algorithm(s)	Accuracy	Task(s)
^7^	2019	Authors used two DL models namely FFNN and SNN to classify attacks in IoT network	BoT-IoT	FFNN, SNN	FFNN: 95.1%	C–D
^8^	2018	Zhou et al., have proposed a model for cyberattack detection in the IoT environment	NSL-KDD	Deep Feed Forward Neural Network + backpropagation	95%	D
			UNSW-NB15		< 95%	
^9^	2018	Authors evaluated the RBM as a ML model to distinguish between abnormal and normal flow traffic	ISCX	RBM	89%	D
^10^	2017	Various ML techniques are used, for detecting intrusions based on the classifications applied on the proposed dataset	Synchrophasor dataset	DNN, SVM, RF, DT, NB	DNN: 79.86% < 80%	C–D
^11^	2019	Authors evaluated the performance of several popular ML methods and a DL model in both multi-class and binary classification for detecting intrusions	TON_IoT	Seven supervised Machine Learning methods	CART: 77%	C–D
^12^	2019	Authors presents an intrusion detection model based on Genetic Algorithm and DBN	NSL-KDD	Genetic Algorithm and DBN	> 99%	D
^13^	2020	Authors conducted a comparative study of seven DL approaches under two new datasets for intrusion detection	Bot-IoT and CSE-CIC-IDS2018	RNN, DNN, RBM, CNN, DBN, DBM, DAE	98.394%	C–D
^14^	2019	Authors aims at detecting intrusion to the IoT	IoTD	GAN	20% higher accuracy	D
^15^	2019	Intrusion Detection System for IoT based on a ML approach	NSL-KDD	DNN	97%	D
^16^	2019	Deep learning-based intrusion detection for IoT networks. Authors adopted Bot-IoT dataset, a newly published IoT dataset, they developed a FNN model (feed-forward neural networks) for multi-class and binary classification	Bot-IoT	FNN	99.41%	C–D
^17^	2020	In the classification, their proposed method performed well. The proposed technique improves the precision of classification of normal and abnormal of the network traffic	UNSW-NB15	AC-GAN	96%	C–D
^18^	2019	They used four different DL models in IoT networks and discussed their result analysis in terms of accuracy	NSL-KDD	MLP, CNN, DNN, AE	DNN: 99.24%	D
			UNSW-NB15		No accuracy value	
^19^	2018	Authors used five ML classification algorithm for the attack classification problem under three datasets	KDD Cup'99	C4.5, KNN, SVM, NB, RF	C4.5: 99.94%	C–D
			NSL-KDD cup		KNN: 99.43%	
			GureKDD cup		KNN: 99.08%	
^21^	2018	Authors have adapted an LSTM to detect cyber-attacks. Their experiment was conducted on two datasets	AWID	LSTM	98.22%	C–D
^22^	2020	Authors used a DL model for wireless IDS	AWID	FFDNN	Binary classification: 99.66% Multiclass: 99.77%	C–D
			UNSW-NB15		Binary classification: 85.48% Multiclass: 74.78%	
^23^	2019	Authors used LSTM network model, to classify an incoming packet is a part of malicious traffic or normal	Mirai-CCU	LSTM	99.46%	C–D
			Mirai-RGU		**100%**	
			ISCX2012		99.99%	
			USTC- TFC2016		99.99%	
^24^	2021	Three different sources were studied for the performance of the proposed IDS	Bot-IoT	RNN	99.912%	D
			CICIDS2017		99.811%	
			Power System		96.822%	
^25^	2019	The authors describe a new IoT dataset that they used for their experiments. Results showed a high accuracy in Binary classification. The prediction output was classified as either normal or attack	BoT-IoT	LSTM	Binary classification: 98%	C
				RNN	Binary classification: 97%	
^26^	2019	Authors provide a survey for security of IoT Networks	–	Neural Network	99%	C–D
^27^	2022	Authors used kernel extreme learning machine (KELM) for classification to detect anomalies in IoT	–	KELM	99.40%	C–D
^28^	2022	Authors aims at detecting intrusion in IIoT using classification and detection methods. They used XGBoost (eXtremely Gradient Boosting) model	TON_IoT	XGBoost	96.5%	C–D
^29^	2021	Ullah et al. proposed a DL model IDS based on a CNN for multicast and binary classifications	–	CNN	99.7%	C–D
^30^	2022	Saba et al. proposed a DL IDS model for anomaly-based IDS based on a CNN technique. The proposed model is capable to detect any aberrant traffic behavior and potential intrusion	NID (Network Intrusion Detection) Dataset	CNN	99.51%	D
			BoT-IoT		92.85%	
^31^	2022	For Edge-Based IIoT Security, Guezzaz et al. uses ML techniques to provide a hybrid IDS	NSL-KDD	KNN and PCA (Principal component analysis)	99.1%	D
			Bot-IoT		98.2%	
^32^	2022	Jamal et al., proposed a methodology for malware attacks classification and detection in IoT network. They used ANN	ToN_IoT	DNN	94.17%	C–D
^33^	2022	For IoT networks, Basati et al., proposed a novel DNN-based NIDS characterized by a lightweight architecture of NN (neural network) based on PDAE (Parallel Deep Auto-Encoder)	KDDCup99, CICIDS2017, UNSW-NB15	PDAE	99.37%	D
^34^	2020	Ali et al., aimed to study the effects of poisoning attacks on DL based IDSs for heterogeneous wireless communications. The experimental results show that their proposed poisoning attack significantly decrease the DNN classifier performance by about 13%-17%	NSL-KDD	DNN	84.14%	D
^35^	2023	Habibi et al., focused on the IoT Botnets attacks detection. They used MLP model which shows high F1-score and accuracy as well as high specificity and sensitivity	Bot-IoT	MLP	98.93%	D

## Research methodology

This section presents a survey on AI for IoT security, in which we will study previous works from 2017 to 2023 by comparing the results obtained. The survey is the only one that collects and compares the results of other related works that experimentally test the performance of one or multiple ML/DL methods in IoT attacks classification/detection and presents the current state of research. This section is based on two processes: a search process and a selection process. The following section will be based on an in-depth analysis process and a synthesis process. In this study, we used the criteria (iot OR "Internet of Things") AND security AND (ai OR "artificial intelligence") AND (IDS OR “intrusion detection system” OR intrusion AND detection) to narrow and limit our research. Afterward, we selected and covered only the well-defined, recent and relevant papers which were published between September 2017 and February 2023, as well as we ignored several papers which are not relate to the purpose of this research, in addition to irrelevant studies. Then, we limited our study by excluding workshop proposals, overviews, theoretical study, abstract-only, research not related to the proposed research questions, and finally we got 28 papers for this survey. So, this work is based on well-defined papers which mean the papers that define the purpose (for IDS or other solution), the used algorithms and gives final results. The obtained results were extracted from the Scopus database. The purpose of the followed methodology used in this survey is to compare the accuracy of the proposed AI techniques to find the best one and obtain some inspiring results by performing an in-depth analysis of selected works published over the last seven years. The objective of this contribution is to choose in the first step the best score (or the perfect accuracy) of the AI methods, then, if the choice of this AI algorithm is for classification or/and detection use. To do this, at first, an analysis on published papers on AI-based IoT security system has been presented. This performance comparison aims us to deduce the algorithm that gives the best results in order to determine which is the effective one. At the end, we present possible challenges of AI with IoT and their future research directions.

The proposed methodology followed in this study is based on a large comparison between previous research concerning the method used, dataset used, the parameter used. A comparison of accuracy rate will be made in order to deduce the best AI technique among them. This paper is a survey of surveys/(related works) which aims at reducing tasks in terms of time. As Table 2 shows, there are wide studies devoted to the IoT security systems including classification and detection, which show and demonstrate the importance and relevance of the topic. Table 2 shows the main results extracted from these recent papers.

In the Table 2 bellow, accuracy is used for performance evaluation. Because, in the literature, several researchers evaluate and test their algorithm using the accuracy metric. Based on the previous section, we will be updated with the new datasets used for IoT security, the techniques used, and the obtained accuracy rate. The performance study of the AI algorithms including ML and DL has been done related to some essential parameters like year of publication, objective of the contribution, dataset(s) used, algorithm(s) used, results obtained in term of accuracy, the treated problem(s) or task(s) in which the algorithm(s) were applied, it includes: (1) C: classification; (2) D: detection; and (3) C-D: Classification and detection of intrusions, anomalies (deviants, outliers) or attacks. Datasets are the collection of data that can be used for training and testing the AI methods. Accuracy is used as the reference value of each algorithm. The bold value is the high accuracy obtained from the literature.

## Results and discussion

This paper presents a performance study and a comparative study between AI algorithms for improving IoT security systems. The goal of this section is to explain clearly and deeply the obtained results. Further, we gathered all these results in order to be explored in our future research. This section comprises of four subsections. It provides a comparison of datasets and AI algorithms for improving IoT security systems. The first subsection provides insights of the analysis and comparison of publicly available datasets that have been applied in IoT security. The next subsection details on the analysis on AI methods for classifying and detecting IoT security attacks. The third subsection proposes a new taxonomy of AI algorithms for IoT security. Finally, the challenges of AI with IoT were discussed. This means that the last two subsections mainly revolve around challenges and applications of AI including machine/deep learning (ML and DL) in the concept of IoT security. The following subsections will provide the insights gained.

Authors use more than one algorithm and compare them on dataset or more datasets. Cross Table 2 contains all these results. It is a comparison table between the different algorithms, the datasets used for the tests by each author. This survey presents the different methods used, the different algorithms used as well as a comprehensive and comparative study on all the algorithms used for the security of IoT systems.

This paper provides a good reference for researchers and readers in the IoT domain to develop new solutions for improving IoT security systems based on AI. In order to execute the conducted survey, we followed the methodology proposed in this work, their purpose is to compare the accuracy of the proposed AI technique to obtain some inspiring results. Based on it, the first observation is that different works might use different datasets and models, i.e., each researcher has its own method of working, and each one of them aims at improving the effectiveness of its obtained results. This means, in this literature review, some surveys have been exploited one dataset to evaluate their model or (more than one model) while others used different datasets for testing their model/method (models). The integration of AI in IoT systems based on many directions of perspective will enable the improvement of IoT security systems.

### Analysis and comparison of publicly available datasets

This subsection provides a comparison of datasets obtained from the literature by critically analyzing the strengths and weaknesses of each dataset. Each dataset has its own features. The aim is to classify the datasets and choose the best for us. Regarding the datasets, choosing recent and publicly accessible datasets on IoT is challenging. Hence, efficient datasets are needed in order to implement the (chosen) AI technique in IoT environment.

The major challenge in developing attack detection is the lack of appropriate IoT datasets. Therefore, in IoT domain, to develop attack detection, we need appropriate IoT datasets. Datasets must be suitable for the IoT systems, which means specifically for IoT, large, new, rich, high quality, updated, publicly available, and very sufficient in order to improve learning and get the best results as well as to increase the security in the context of IoT networks. Data/dataset preprocessing using the Python environment (the universal programming language) is an important phase for identifying features and cleaning data. The learning base must be rich enough. Therefore, to improve learning, and get a high recognition rate as well as to obtain complete results, a large and very sufficient dataset is needed. Indeed, these datasets must really contain attacks on IoT systems.

**Figure 7:**
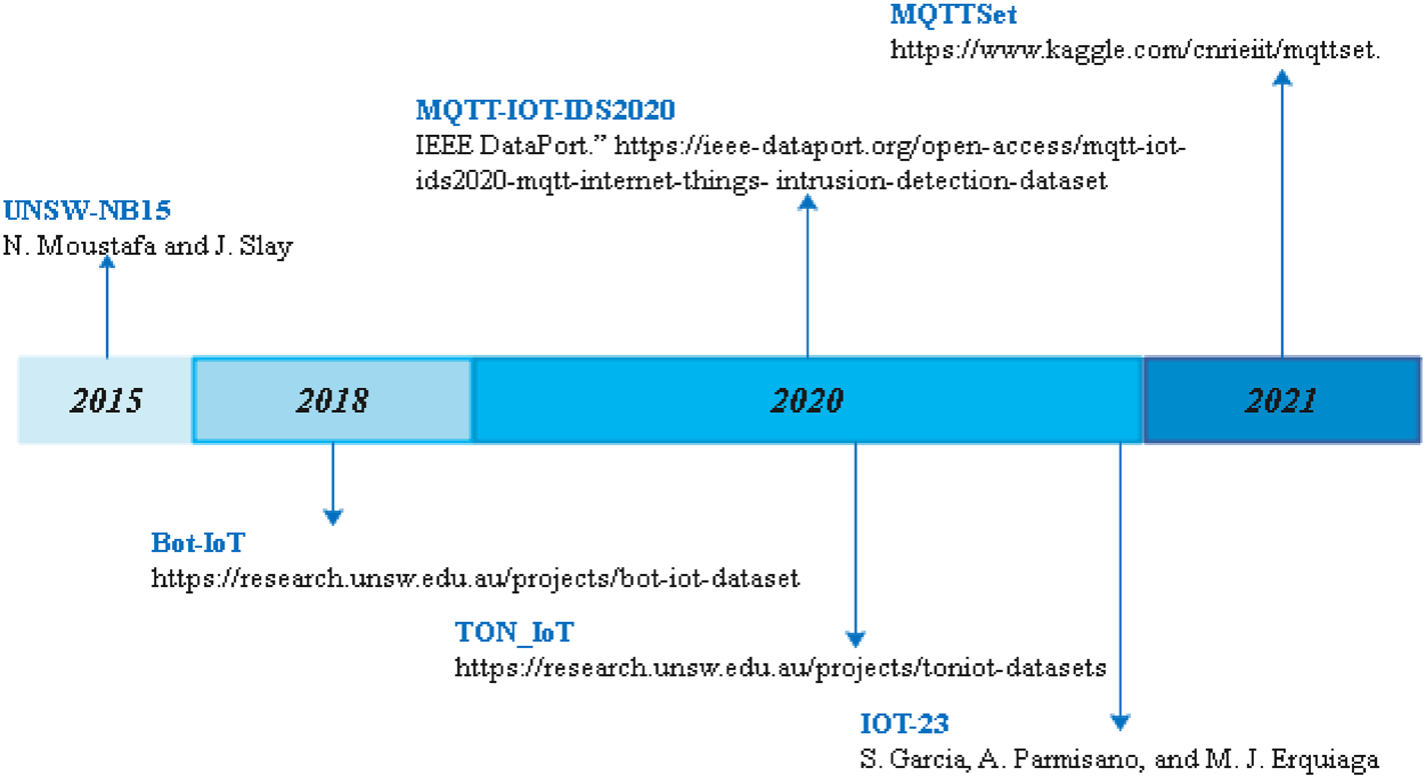
Typical datasets for IoT security obtained from the different related works.

The used datasets like KDDCUP 99^19,33^, ISCXIDS2012^21^, NSL-KDD^8,12,15,18,19,31^, and CSE-CIC-IDS2018^13^ are not IoT-oriented. Whereas, datasets such as Bot-IoT^7,13,24,25,30,31,35^, TON_IoT^11,28,32^, MQTT-IOT-IDS2020^36^, MQTTSet^37^, and IOT-23^38^ are the typical IoT datasets. The following Fig. 7 displays typical IoT datasets obtained from the different related works. Indeed, the UNSW-NB15^8,17,18,22,33^ dataset is showed in the Fig. 7 because it is composed of some IoT traffic. The goal is to choose a dataset among the datasets found. Based on Table 2, works^8,12,15,18,19,31,33^ were based on old resources, which is outdated, the sources must be updated. Recently, TON_IoT and Bot-IoT are used a lot^7,11,13,24,25,28,30–32,35^. Moreover, the Bot-IoT dataset is widely used a lot because it is the first created (2018) (see Fig. 7). Based on Tables 2 and 3 we can conclude that:

- Between 2017 and 2019, there is an orientation to KDDCUP 99, NSL-KDD which are not compatible to IoT environment. Several authors use them^8,12,15,18,19,31,33^;
- Between late 2020–2021: Generation of new datasets that are 100% compatible with the IoT environment

**Table 3:** A comparative study between the most recent IoT datasets.

IoT datasets	Year	Attack categories	Availability	Authors	Application	Developer	Web link	Labeled data	Size	Drawbacks
MQTT-IoT-IDS2020^36^	2020	MQTT brute-force attack, UDP scan, Sparta SSH brute-force, and Aggressive scan	Yes/Free/ Downloadable	H. Hindy, C. Tachtatzis, R. Atkinson, E. Bayne, X. Bellekens	IoT cybersecurity	IEEE Data Port	https://ieee-dataport.org/open-access/mqtt-iot-ids2020-mqtt-internet-things-intrusion-detection-dataset	Yes	Approx. 1.65 GB	MQTT-IoT-IDS2020 exclusively focused on the MQTT protocol
MQTTSet^37^	2021	DoS, SlowITe, Flooding, Bruteforce, and Malformed data	Yes/Free/ Downloadable	I. Vaccari, G. Chiola, M. Aiello, M. Mongelli, and E. Cambiaso	IoT cybersecurity	–	https://www.kaggle.com/cnrieiit/mqttset	Yes	10 GB	MQTTSet only applicable on the MQTT protocol
IOT-23^38^	2020	Nine attack categories	Yes/Free/ Downloadable	S. Garcia, A. Parmisano, and M. J. Erquiaga	IoT cybersecurity	Avast, AIC group, CTU	https://zenodo.org/record/4743746#.YXeyAhyEZPY	Yes	21 GB	IOT-23 lacks some kinds of attacks
Bot-IoT^39^	2018	DDoS, DoS, reconnaissance, and theft	Yes/Free/ Downloadable	K. Nickolaos, M. Nour, E. Sitnikova, and T. Benjamin,	IoT botnet	Cyber Range Lab of the University of New South Wales (UNSW) Canberra Cyber	https://www.unsw.adfa.edu.au/unsw-canberra-cyber/cybersecurity/ADFA-NB15-Datasets/bot_iot.php	Yes	69.3 GB	Bot_IoT lacks some kinds of attacks and does not contain the different types of the applications of IoT
TON_IoT^40^	2020	DDoS, DoS, Scanning, password cracking attack, XSS, data injection, ransomware, backdoor and MITM attack	Yes/Free/ Downloadable	N. Moustafa	IoT/IIoT cybersecurity	ACCS (Australian Centre for Cyber Security’s) Cyber Range Lab	https://ieee-dataport.org/documents/toniot-datasets	Yes	67 GB	–

According to^25^, existing datasets, introduce various challenges, like poor reliably labeled data, a lack of attack diversity such as redundancy of traffic, botnet scenarios, and missing ground truth. However, the Bot-IoT is a new dataset addresses the aforementioned challenges. The BoT-IoT dataset was created in 2018, specifically for IoT systems, it comprises both attack traffic and legitimate. DoS (HTTP, TCP, UDP), service scan, DDoS (HTTP, TCP, UDP), keylogging, and data exfiltration, are the included attacks. The collected dataset was from a realistic representation of an IoT network, it includes botnet and normal traffic. It contains over 72 million records of network activity/traffic. These records were collected from a simulated IoT environment^11^. However, this dataset does not include sensor readings of IoT devices. The TON_IoT dataset contains heterogeneous data sources gathered from the Telemetry data of IoT/IIoT services, and the Operating Systems logs as well as Network traffic of IoT network, that were collected from a realistic representation of a medium-scale network designed at the Cyber Range and IoT Labs at the UNSW Canberra. It includes nine types of cyber-attacks namely DDoS, DoS, Scanning, password cracking attack, Cross-site Scripting (XSS), data injection, ransomware, backdoor and MITM (Man-in-The-Middle)^11^. The TON_IoT has various advantages and new properties which are currently lacking in the existing (state-of-the-art) datasets^11^:

- TON_IoT has various attack and normal events for different IoT and IIoT services;
- TON_IoT includes heterogeneous data sources;
- TON_IoT was collected from a testbed with a realistic representation of a IoT architecture for communicating Edge, Fog and Cloud layers.

The MQTT-IoT-IDS2020 dataset (MQTT internet of things intrusion detection dataset) was built by a simulated MQTT network. It includes twelve sensors, a simulated camera, a broker, and an intruder/attacker. This dataset contains five labels, four labels are attacks as shown in Table 3, and the fifth is for normal. A recent IoT dataset specific to the MQTT protocol and adopted for IoT networks was created in 2021, called MQTTSet, it includes an MQTT broker and eight IoT sensors in a smart home like humidity, door opening/closure, temperature, CO-Gas, smoke, fan status, light intensity, and motion at different temporal intervals. IOT-23^38^ was concerned on DNS traffic in the IoT context.

The novelty focuses on introducing recent IoT datasets. The Table 3 presents a comparative study between the most recent IoT datasets (created between 2018 and 2021) which containing IoT traces. Bot-IoT dataset did not contains a variety of types of attacks. KDDCUP 99, and NSL-KDD datasets are not for IoT systems and outdated. As a consequence, the UNSW-NB15 dataset is eliminated in the Table 3, because it was not new, it was created in 2015 by N. Moustafa and J. Slay^41^, and it was not an IoT dataset, it is for a general purpose, as well as it does not include specific characteristics of IoT/IIoT app. Only network features that were extracted from MAC (Media Access Control) layer from 802.11 wireless network are included in the AWID dataset. KDDCUP 99, NSL-KDD, UNSW-NB15, AWID, Bot-IoT, and ISCX do not include IoT telemetry data.

Table 3 only shows the most publicly available cybersecurity IoT datasets and not network security datasets. Table 3 compares the datasets analyzed during this current study and illustrates the comparison criteria used: (1) Year, (2) Attack categories, (3) Availability, (4) Authors, (5) Application, (6) Developer, (7) Web link, (8) Labeled data, (9) Size, (10) Drawbacks. Based on Table 3, we can say that we found only few datasets for IoT named MQTT-IoT-IDS2020, MQTTSet, IOT-23, Bot-IoT, TON_IoT. These IoT datasets are publicly available, free, downloadable via their persistent web link to datasets and not on reasonable request of their corresponding author (i.e., data are not public). If the data are not public, then, it will be available from the corresponding author on reasonable request. No permissions are available on request by the Publisher for analyzing these datasets as shown in Table 3.

Among the comparison, we can conclude that TON_IoT is a newly generated and publicly available dataset in IoT and IIoT networks for evaluating an IDS and it has many advantages compared to other existed datasets. The TON_IoT dataset contains IoT/IIoT service telemetry. This comparison found that TON_IoT has a better advantage than older datasets. This is a recent source, the sources must be updated and not outdated.

In conclusion, the majority of IoT datasets that have recently been published were created to test IoT network-based IDSs. However, they do not have the actual data (i.e., measurement/telemetry data) generated from sensor readings; instead, to detect attacks on IoT networks, they primarily comprise flow/packet-level information, or a combination of both^11^. This dataset (TON_IoT) includes 7 IoT representing Thermostat, Fridge sensor, Motion light sensor, Garage door, Weather, GPS (Global Positioning Sensor) sensor, and Modbus. Based on various IoT device data, this dataset offers an accurate environment for an IDS for IoT devices.

For training and evaluation, such IDSs require an up-to-date and representative IoT dataset. The evaluation of AI methods including the intrusion detection methods plays specific role. Indeed, the evaluation of the efficiency of IoT security methods and the accuracy are related to the used IoT datasets which must reflect real-world for IoT applications. The use of IoT-related datasets that reflect real-world IoT applications plays a vital role. However, benchmark IoT and IIoT datasets for evaluating IDSs-enabled IoT systems are lack^11^. In addition, there is a lack of availability of real-world datasets for IoT and IIoT applications^11^.

### Analysis on AI methods for classifying and detecting IoT security attacks

Concerning AI algorithms, the chosen model is the LSTM model that is a DL method, because it achieved the high performance in term of accuracy, as well as it was achieved the highest accuracy in several works as example, we cite^23,25^ than other techniques. After this analyzed works, the concluded results show that LSTM is the best one for classifying the incoming packet into the abnormal and normal state^23^. The evaluation results of^23^ showed that the proposed framework on four datasets including (1) IoT data set collected on their Mirai botnet (Mirai-CCU), (2) USTC-TFC2016, (3) ISCX2012, and (4) IoT data set from Robert Gordon University (Mirai-RGU), can achieve near 100% accuracy as well as precision in detecting malicious packets. In other words, the best AI algorithm prediction accuracy was 99.99% and going to 100% on the four datasets. On the other hand, the LSTM algorithm can perform the classification with nearly 100% accuracy^23^. So, with this model, they obtained an accuracy of 100%, a precision of 100%, a recall of 100%, a F-Score of 100%, and a false positive rate of 0%. In other words, the obtained results are promising not only in terms of accuracy but also in multiple metrics like precision, recall, F-Score and false positive rate. The second observation is that the highest and the perfect accuracy gained was by the LSTM model.

Authors of^11^, demonstrated that the LSTM model can be formulated as a classification problem in a supervised manner to be used for attack detection. To sum up, and based on^13,22^ as well as several searches, I observed that the DL-based classification approaches is gaining the best classification accuracy or performance than classical ML approaches for both the binary classification and the multiclass classification. The results show that LSTM outperforms other models. Finally, the LSTM outperformed other Artificial Intelligence algorithms, especially, the Deep Learning (DL) algorithms. Whereas, the lowest accuracy score was 54% which has done by NB (Naïve Bayes) method according to^11^. The third observation is that DL-based classifiers/models are more accurate than classical ML methods. i.e., classical ML methods lack accuracy, scalability, and robustness. In addition, memory requirements^21^ and detection speed of DL are better compared to those of classical ML. The main drawback of traditional Machine Learning and many Deep Learning techniques is the lack of memory^21^ to recall previous events. This limitation is solved by RNN.

The next question that can be asked is as follows: can we use the LSTM (Long Short-Term Memory) technique to classify and/or detect attacks and intrusions in the IoT security systems? based on^13^, DL techniques, especially Neural Networks types (NNs) can be used for attack classification and anomaly detection tasks in IoT. In addition, in^25^ the LSTM model was evaluated on binary classification on two datasets and it can also be used for anomaly detection^21^ have used LSTM for attack detection and considered two datasets utilized for multiclass and binary classifications. (Hwang et al.^23^) used it for classifying malicious traffic, they also suggested an IDS for detecting malicious network traffic. Besides, in^11^, it can be used for attack detection. The fourth observation is that the AI is a good tool to adopt for improving IoT security system, especially, the LSTM-based DL approach that can be used for both tasks (classification and detection). For instance, in the work^25^, the LSTM model has been used for anomaly detection task, whereas, in^13^, it has been used for anomaly detection and attack classification tasks. Many surveys try to evaluate the performance of AI algorithms, the best and the highest accuracy reached was under a LSTM technique. But all of rest (including ML and other DL techniques and algorithms) the accuracy doesn’t reach 100%.

Regarding the performance of AI techniques, the methodology followed in the Table 2, shows that all accuracies rates are more than 74.78%. The accuracy of the LSTM model is about 99.99%. So, if we compare it with the other results of the literature works, we can say that, the LSTM method has been successfully applied for detecting anomalies and intrusions as well as attack classification or classifying malicious traffic, which also means that the LSTM model classify the incoming packet into the abnormal and normal state.

As a result, LSTM is classified as the best algorithms among the AI techniques, in spite the fact that it recorded high accuracy. This choice of the model is justified by a comprehensive analysis and a performance evaluation of the existing AI methods used especially for IoT security systems. This research has been made in order to improve the IoT security systems. For this reason, I precise the task (classification and/or detection) in which we can apply the LSTM algorithm in IoT systems. In other words, choosing a wrong AI technique could lead to loss accuracy and effectiveness. Indeed, choosing a wrong dataset will produce incorrect and erroneous results. The pre-processing of the chosen dataset (including identifying features and cleaning data) is also important for improving the prediction accuracy in the IoT security. Additionally, in the fifth observation, I concluded that the DL techniques is more suitable for attack classification and anomaly detection tasks compared to ML in the IoT environment. In other words, DL-based algorithms have better classification and detection accuracy as compared to the traditional ML models for IoT attack classification and detection. To compare the performance of AI algorithms, many studies have been conducted using different approaches, datasets, and methodologies for different use cases.

After obtaining these promising results, we can conclude that the DL based LSTM technique can be an ideal solution not only for attack classification, but also for attack detection. The results and findings clearly show that the LSTM algorithm is the best way to resolve both classification and detection problems in the IoT domain. The LSTM method achieved the best performance in the papers^11,13,21,23,25^. Through these previous experiments, we concluded that LSTM algorithm delivers best results than other AI classifiers in terms of accuracy for classifying and detecting attacks and intrusions in IoT systems. According to these studies the LSTM method allows to achieve the best accuracy rate, so, the classification and the detection of IoT attacks and intrusions by LSTM leads to good results. In terms of a comparison between the different ML and DL techniques, we compared the AI algorithms to deduce the one which gives us the suitable/best results, and we got the best results with the LSTM. In other words, Table 2 has been showed that the LSTM algorithm is more accurate than the other AI algorithms used in the literature. LSTM's accuracies range from 98 to 100%, for the BoT-IoT, AWID, Mirai-CCU, Mirai-RGU, ISCX2012, USTC-TFC2016, and CSE-CIC-IDS2018 datasets.

In the second step we will detail the proposed new taxonomy of AI algorithms for IoT security. Indeed, it analyzes these AI algorithms by giving a summary comparison, in order to have a comparative study between the most known classification techniques for IoT attacks. This study aims at illustrating the applications of some of the best-known ML/DL approaches to a classification problem of IoT attacks by covering and detailing the advantages and disadvantages of each one. This survey, covers and combines several technologies and methods for securing IoT systems.

### Proposed new taxonomy of AI algorithms for IoT security

In this subsection, I try to propose a general taxonomy to summarize the supervised classification algorithms used in IoT security. The objective is to present the most known method for classifying attacks in IoT security. In this subsection, the suggested taxonomy of supervised classification techniques can be applied in IoT security. Thus, in this paper, we provide an overview of AI techniques and its applications in IoT security, based on that we propose a novel taxonomy of AI-based IoT security to improve the success of AI techniques in IoT security systems. The aim is to explore ML and DL for IoT attack classification and present a new taxonomy of them, with also a comparison between these classifiers.

A detailed existed taxonomy is given by^42^ summarizes a general outline of taxonomy of ML algorithms, it contains 63 algorithms categorized into 11 classes namely DL techniques, NN, Bayesian, DT, Regression, Clustering, Regularization, Rule System, Instance based, Ensemble and Dimensionality Reduction. Despite its advantages, this taxonomy has not got a clear vision. Further, this taxonomy lacks some important algorithms of AI techniques, and the CTM method will also be categorized.

The idea is to summarize the eleven classes existing in^42^ in only six classes and each class is divided into subclasses. Moreover, it is still lacked the most popular ML algorithms like SVM, Reduced Error Pruning Tree (REPTree), Multilayer Perceptron (MLP), Feedforward NN (FFNN), Stochastic Gradient Descent. So, these algorithms must be added in ML subclasses, but, Recurrent Neural Networks (RNNs), Long Short-Term Memory Networks (LSTMs), Generative Adversarial Networks (GAN), Feed forward Deep Networks (FDN) can be categorized as DL techniques.

The taxonomy existed in the image/figure^42^ lacks some important and known algorithms like SVM, that we will be categorized in Instance based. Besides, Recurrent Neural Networks (RNNs) and Long Short-Term Memory Networks (LSTMs) will be classified in DL techniques. Furthermore, Multilayer Perceptron (MLP) and Stochastic Gradient Descent will be categorized in ANN. Moreover, GAN and FDN can be classified according to this survey in DL techniques. Otherwise, the study in^43^ categorized FFNN in NN techniques. Thereafter, REPTree is another DT learner used for classification, it is aimed at building simpler and faster tree models using information gain for splitting.

**Figure 8:**
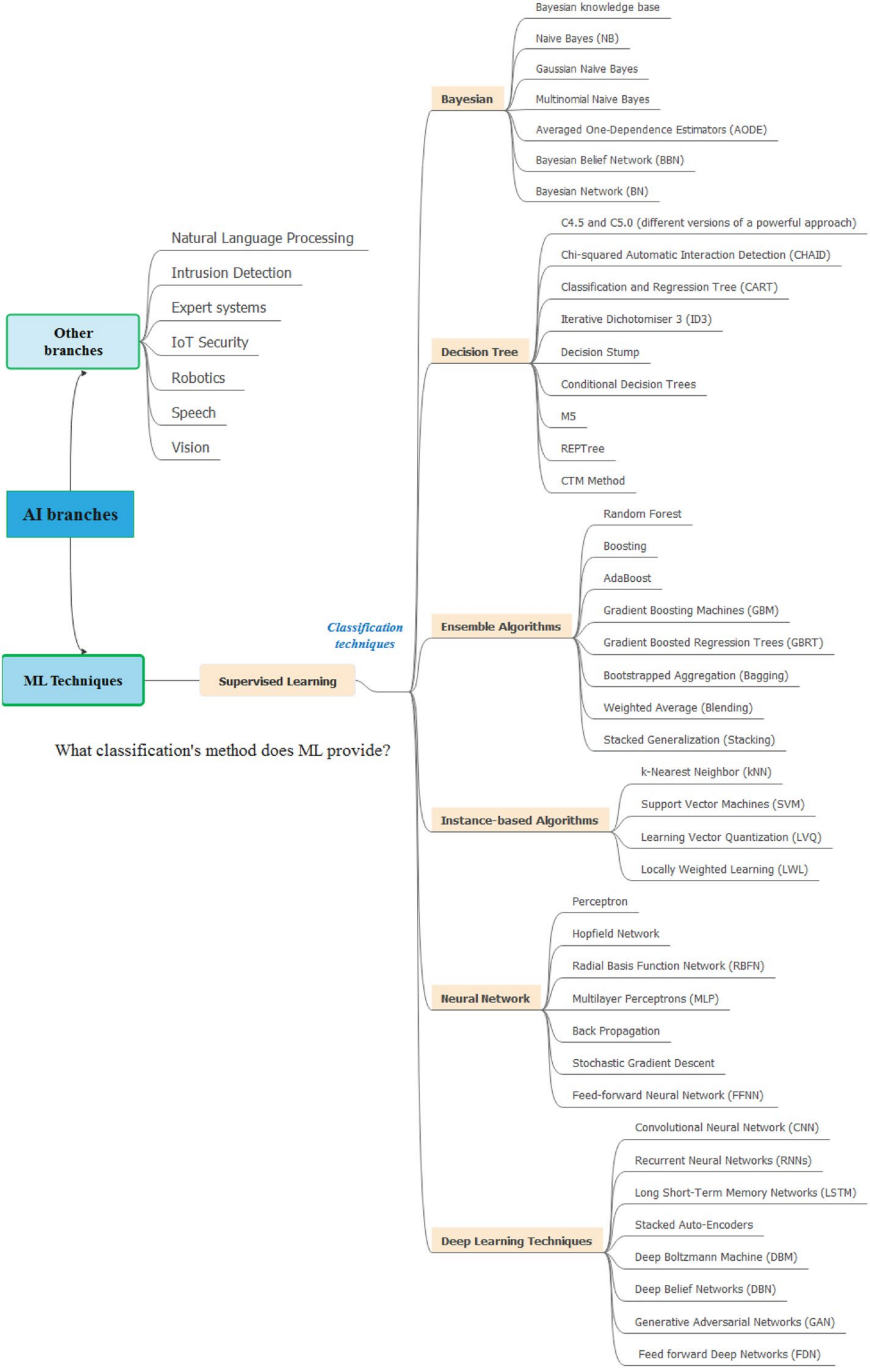
Taxonomy of AI techniques used for classification task for IoT security.

Additionally, the Classification Tree Method^1,3^ (CTM method) does not classified by any authors in the literature review. So, for this reason, I suggest to classify CTM method in the DT class (see Fig. 8). A brief taxonomy of AI subsets is proposed in Fig. 8 in which, six major categories of classification methods are mainly presented: Bayesian algorithms, Decision Tree, Ensemble algorithms, Instance-based algorithms, Neural Network and DL techniques. Each class of classification techniques is divided into several algorithms.

In this work, we eliminate the Self-Organizing Map (SOM) from the proposed taxonomy, because, it is an unsupervised ANN learning. However, DL techniques are not limited only to CNN, DBM (Deep Boltzmann Machine), DBN (Deep belief network), SAE (Stacked Autoencoder), but there are others techniques like RNN, DNN, AE (Autoencoder), RBM (Restricted Boltzmann machine), GAN. In this context, this class can be divided into two subclasses, the first one is supervised DL techniques which contains DNN, RNN, CNN, DBN. The second one is unsupervised DL techniques including GAN, RBM, AE.

In addition to this, ML algorithms can be grouped into three major categories, namely, supervised learning, unsupervised learning and reinforcement learning, for each class there are subclasses. This subsection will limit its research in only supervised learning, because the classification techniques belong it. In supervised learning there are two subclasses named classification and regression algorithms. Unsupervised learning contains clustering and dimensionality reduction.

In this context, we are interested on supervised learning and more precisely on supervised classification algorithms that can be used to classify and detect attacks. Most supervised learning algorithms attempt to find a model (a mathematical function) that explains the relationship between input data and output classes. This study aims at illustrating the applications of some of the best-known ML approaches to a classification problem of IoT attacks by covering and detailing the advantages and disadvantages of each one. Then, in the following, we displayed this existing classification methods/classifiers with their working principal, each classifier has its own strength and limitation. Among them we cite the most popular classifier: k-NN, SVM, DT, BN, RF, etc.

Several recent surveys in this area include^44–53^ have been conducted on several aspects of AI and its integration with IoT security, however, most of these studies are theoretical. There is no promising work on integrating AI models into real IDS systems for IoT in order to eliminate both false negatives and false positives, as well as to make a real test and evaluate their models with real attacks with real network traffic and deploy it in reality (in real IoT environment).

The study^49^ also covered some aspects of DL and big data for IoT security. There are researches that work on IoT attacks classification or/and detection using different AI algorithms or use other methods such as statistical models to detect anomalies in the IoT system. Most of the current work in the field of AI can be called “narrow AI”^48^. Narrow AI means that AI-based solutions which address and solve a specific problem. Thus, the opportunities and possibilities of both AI and IoT can advance^48^ when they are combined. The IoT needs to rely on AI because it is impossible for a human being to find information in the data generated by the IoT^48^. Without AI, the data generated by the IoT remains useless. Furthermore, if a new pattern is detected in the data, the machine will be sufficiently capable of learning on its own, which would be impossible on any non-AI IoT system to do^48^.

To have a relevant AI classification technique, we also aim to make a comparative study of the AI classification techniques with other classification method proposed by^1,3^. There are many researches to classify IoT attacks. Each author/researcher has its own method of working. Each one has different techniques in classification and/or detection. There are researches that work on classification or/and detection using different intelligent ML or DL algorithms or use other classification method such as the CTM method (Classification Tree Method). These parts of AI are discussed in the following. This present survey focuses on presenting the strengths and limitations of each approach that can be used for classification of IoT attacks. It will also provide an overview of the most popular ML and DL algorithms to get an idea of the supervised learning approach used in classifying IoT attacks. The following subsections will demonstrate different types of classification learning, including ML (KNN, SVM, DT, NB, etc.) and DL (CNN, RNN, etc.). To do this, we will mention in detail the existing methods by describing briefly their advantages/disadvantages with also the IoT security application of it. A taxonomy of AI techniques used for classification task is shown in Fig. 8. This proposed taxonomy can help researchers address AI techniques in the context of classifying and detecting attacks and intrusions to improve IoT security systems.

This section studied different AI techniques. The goal of it is to make a general comparative study due to the lack of updated work on comparing different ML and DL techniques. In this work, three different classification algorithms are provided which are: CTM method, ML and DL techniques. The main objective of this systematic research is to present the AI algorithms used in classification tasks. This survey takes a deeper look and analysis at how AI algorithms are being used to classify attacks in IoT domain. In addition, the following tables summarize what is detailed as advantages and disadvantages. Consequently, the goal is to make a synthesis and choice of algorithm that I found better compared to others and can be exploited in IoT security in general and IoT attacks classification and detection in particular. So, AI algorithms are important in securing IoT systems. In the following, we make a comparative study for different AI classification techniques used for IoT security purpose. Thus, we critically deal with the AI algorithms and provide their applicability in IoT security systems.

The CTM method^1,3^ classifies the various attacks on IoT security, and embedded systems to automatically generate test cases and selects the relevant test cases. Indeed, it is a formal method for graphically representing test cases. It provides a systematic procedure for creating problem-specific test cases. Its basic idea is to ignore test data and separate the input of the test into several classes/subsets according to the aspect that is relevant by the tester.

KNN (K-Nearest Neighbour) method is easy to apply, simple, cheap, and it is used in malware detections, intrusion detection, and anomaly detection in the IoT, DoS/DDoS attack detection^47,52^, authentication of an IoT element^50^. The principle of the k-NN algorithm is based on the choice of the class from the classes of the nearest neighbors, that is to say, it is about making decisions by looking for one or more similar cases in the learning set. The trick is to determine the similarity between the data instances. k-NN captures the idea of similarity (also called distance proximity or closeness). This method requires choosing a distance (Euclidean distance) and the number of neighbors to take into account.

SVM (Support Vector Machine) has a high level of accuracy that makes it suitable for IoT security applications such as intrusion detection, smart grid attacks, malware detection, DoS/DDoS attack detection^47,52^, authentication of an IoT element^50^. However, the disadvantage of using this technique is that sometimes it is difficult to use an optimal kernel function^52^.

DTs (Decision Tree) are used as a classifier in security application such as intrusion detection^50^, DDoS, Device authentication; DoS/DDoS attack detection^47,52^, authentication of an IoT element^50^.

NB (Nave Bayes) is used in IoT for anomaly detection and detecting intrusion in the network layer. It can also be used for device authentication^47,50,52^. NB is a probabilistic model based on Bayes theorem. The Bayesian network is a probabilistic graphical model for knowledge acquisition, enrichment and exploitation.

EL (Ensemble Learning) is used for intrusion detection, malware detection, and anomaly detection^47^. It is well suited for solving most problems because it uses several learning algorithms. However, EL has high time complexity compared to another single classifier^52^.

RF (Random Forest) uses a couple of DTs to create an algorithm in order to obtain an accurate and strong outcomes estimation model. It is used in anomaly detection, DDoS attack detection, Device authentication; DoS/DDoS attack detection; Intrusion detection; Malware detection, unauthorized IoT devices identification in network surface attacks^47,52^.

NN (Neural Network) techniques reduce network response time and therefore increase IoT system performance^47^, detecting DoS attacks in the IoT networks. However, the disadvantage of the use of NNs resides in the” black box” aspect of NNs, i.e., only the overall decision appears at the network output, without understanding and having information about what is behind and the way that this decision was. Which means that we cannot assume the performance of NNs. Now, the NNs are applied for all kinds of applications in multiple fields. The NN can modify itself based on the actions results, which enables the resolution and learning of problems without any conventional programming. A NN is defined by: (1) The number of layers; (2) The number of neurons per layer; (3) The activation functions; (4) The set of all the weights relative to the neurons.

ANN (Artificial Neural Networks) is one of the most widely used algorithms in ML. ANN can be used for Security of IoT networks^50^, Anomaly/Intrusion Detection^44^, DDoS attack detection. The ANN is composed of 3 layers (input, hidden and output). Each layer consists of one or multiple neurons. If ANN was trained with updated^52^ datasets, it performed better in DDoS attack detection.

CNN (Convolutional Neural Networks) and RNN (Recurrent Neural Networks)^52^ can be used for device authentication, intrusion detection; malware detection, DoS/DDoS attack detection. CNN is a supervised learning, which is also a variation of the regular NNs. CNNs also known as convolution nets (ConvNets) are a category of NN (Neural Networks). It’s composed of an input layer, many hidden layers between the input and the output and an output layer. i.e., it is composed of an input layer, followed by one/more convolutional layers, a pooling layer, one/more fully connected layers, finally an output layer. The general CNN architecture consists of convolutional, pooling and fully connected layers. Convolutional network^49^ can be used for malware detection, privacy of an IoT element^50^, security of mobile networks^50^.

RNN (Recurrent Neural Networks) can be used for automatic natural language processing, intrusion/anomaly detection and speech recognition. RNN is considered as supervised or unsupervised learning. It is a multilayer NN with the previous set of hidden unit activations feeding back into the network along with the inputs. RNNs have a memory which captures information about what has been calculated so far. It is for the discipline of automatic NLP (Natural Language Processing). It consists of an input, one/more hidden layers, and an output layer. Each composed of one/more recurrent neurons. The main drawback of traditional Machine Learning is the lack of memory^21^ to recall previous events. RNN solves this limitation/drawback by maintaining loops from current to previous states^21^ to enable information persistence.

LSTM (Long Short-Term Memory) LSTMs are a supervised learning, which is a special of RNN, they are capable of learning long term dependencies. It usually consists of gates and memory cells^11^. Using different gate units in LSTM can solve the problem of gradient vanishing or explosion^11^ caused by memory loss for long-term sequences. For the purpose of detecting attacks, the LSTM model can be developed/formulated as a problem of classification in a supervised manner^11^.

MLP (Multi-layer Perceptron): consists of an input, several hidden layers, and an output layer. Each layer consists of one/more nodes. The input layer receives the signal while the output layer performs a prediction on input. The hidden layer(s) is the true MLP’s computational engines. It is trained in supervised learning way/manner with the BP (back-propagation) algorithm. In other words, for training, the MLP uses backpropagation technique which is a supervised learning technique. In the MLP with backpropagation, neurons (nerve cells) of a layer are linked to all the neurons of the adjacent layers. To solve a problem, the implementation of a MLP algorithm needs the identification of the best weights applicable to each of the inter-neuronal connections through a backpropagation technique. MLP can be used to classify network traffic in IoT backbone network.

RBM (Restricted Boltzmann Machine) is a kind of ANN that can be used for intrusion detection^49^. It is able to represent and solve difficult problems^49^. RBM consists of two types of processes, learning and testing. In the learning phase, a huge amount of examples of desired inputs and outputs are given to create the RBM structure where a general rule is learned for mapping inputs to outputs. In the testing phase, the RBM produces outputs for new inputs while adhering to the general rule obtained during the learning phase.

DBN (Deep Belief Network) is a type of DNN (Deep Neural Network) with several hidden layers that consist of RBM layers. Connections are directed between layers with the exception of the units of each layer. The DBN contains^13^ a layer of hidden units and a layer of visible units. The layer of hidden units learns to represent features and the layer of visible units represents the data^13^. The DBN is a generative graphical model(s) with multiple hidden causal variables. A DBN can be used to fabricate the FFDNN (feed-forward deep neural network) for the IoT^13^.

DAE (Deep Autoencoder) can be used for IoT botnet attack detection^49^, cyber security intrusion detection^13^. An autoencoder is composed^13^ of the decoder and the encoder, it is a type of ANN. DAE is a NN with more than one hidden layer^54^. DAE extracts the internal relationship of the data by learning the optimal network parameters^54^ that will result in an output similar to the input as much as possible.

GAN (Generative Adversarial Network) GANs are an unsupervised learning algorithm which uses two NNs named "Generator" and "Discriminator" opposed to each other. In other words, the GAN combines two NNs, one of which generates/creates the objects, while the second estimates them. The first network is known as G, the Generator, while the second one is known as D, the Discriminator. GAN can be employed for Intrusion detection^55^, and security anomalies^55^.

As shown this section should be detailed, it makes a summary and tool choice that I find better compared to others and can be exploited in IoT security. For that I put the advantages and disadvantages in several tables, to be clearer and more organized. These tables summarize what I have detailed as advantages and disadvantages. In the comparison between CTM method, AI algorithms, previously detailed and shown in Tables 5 and 6, we mainly focus on the tasks, the case study, the strengths and the weaknesses (see Tables 4, 5 and 6).

**Table 4:** Advantages and disadvantages of CTM Method for IoT security.

	Tasks	Case study	Strengths	Weaknesses
CTM Method	Classification	Classifying IoT attacks^1,3^	CTM method is a systematic approach and representative test cases It also has lower complexity Easier to understand Graphical representation of test relevant aspects^56^	There is no strict guidance or algorithm for selection of test relevant aspects^57^

**Table 5:** A comparative analysis between AI classification methods limited to IoT security.

AI Algorithm	Description	Application/problem in IoT security systems	Advantages	Disadvantages
KNN	The principle of the k-NN algorithm is based on the choice of the class from the classes of the nearest neighbors, that is to say, it is about making decisions by looking for one or more similar cases in the learning set. The trick is to determine the similarity between the data instances. k-NN captures the idea of similarity (also called distance proximity or closeness)	KNN is used in anomaly detection, malware/intrusion detection in IoT, Security of IoT Networks, False Data Injection, detect DDoS in IoT, Impersonation Attacks, Authentication of an IoT element	Simple and easy to apply	KNN is a time-consuming to identify missing nodes which pose a challenge in terms of accuracy, Memory limitation
SVM	SVM is used for regression and classification, it is a supervised ML technique with low computational complexity. Furthermore, the original principle of this method is simple, it consists in seeking a surface of decisions or hyperplane in order to separate two classes	Authentication of an IoT element Intrusion/malware detection, Malware analysis in IoT, Smart grid attacks, Detect DDoS in IoT, Abnormal Behavior, and Data Tampering, Security of Mobile networks	SVM has a high level of accuracy which makes it well suited for security applications in IoT^52^	It is difficult to use an optimal kernel function^52^
DT	It is a type of ML that is mostly used for classification problems. The best-known methods for automatically building DT are the ID3 and C4.5 algorithms	Authentication of an IoT element, Intrusion detection, Detect DDoS in IoT, Detection of suspicious traffic sources,	Easy to implement, Simple construction, Handling large data samples^52^	DT requires a large space to store data because of its large construction^52^; High complexity
NB	NB is a probabilistic model based on Bayes theorem. The Bayesian network is a probabilistic graphical model for knowledge acquisition, enrichment and exploitation	In IoT, NB is usually used to detect intrusion in the network layer, Anomaly Detection Security of an IoT Element^52^	Simple to understand, requiring less data for classifications, easy to implement^52^	Bayes classifiers are less accurate; Storage of training samples
EL	EL combines heterogeneous/ homogeneous multi-classifier to achieve an accurate result	Anomaly/malware detection, Intrusion detection, Authentication	It is well suited for solving most problems	High time complexity
RF	It’s an ensemble (a set) of DT. The goal of this category is to group all weak classifiers to form a strong classifier;	Detect DDoS in IoT, Malware analysis in IoT, anomaly detection, Intrusion/Malware detection^52^ Unauthorized IoT devices identification in network surface attacks	RF classifier handles the missing values and maintains accuracy for missing data	It needs more training data sets to create DTs which identify sudden unauthorized intrusions^52^
NN	A NN consists of neurons connected through weighted connections. The NNs are an artificial method for addressing reasoning and learning problems. Different types of NNs are used to improve the classification (ANN, CNN, RNN)	Detect DDoS in IoT, Increase IoT system performance, NN is being used to detect the intrusion attack	NN techniques reduce the network response time and subsequently increases the performance of the IoT system	NNs are computationally complex, Black box, NNs are hard to implement in a distributed IoT system
ANN	It is one of the types of NN. The functioning of ANN is inspired by the neurons of the human brain. The ANN network can do the following: Learning/Training, Classification and Prediction. The ANN was exploited as a mechanism of classification, detection, clustering, and diagnostic	Anomaly/Intrusion Detection, Security of IoT networks, In IoT systems, ANN techniques were used to train the machines for anomalies detection^52^ DDoS attack detection, Malware analysis in IoT Classification and Detection of Ransomware	ANN is much less complex compared to others, It gives good results	ANN requires a learning step/ phase that takes time
CNN	CNN is a deep discriminative model. It is one of the types of NN. CNN is DL model. It consists of multiple hidden layers like convolutional, pooling, fully connected and normalization layers. The most important layer is convolutional layer	Malware analysis in IoT Classification and Detection of Ransomware Intrusion detection	CNN requires less time for training, Fast recognition of the nature of attack	CNN requires high computational power;
RNN	RNN is a deep discriminative model. It is one of the types of NN. RNNs are derived from FFNN with loops and memories	Malware analysis in IoT Intrusion/anomaly detection RNN is used to train the IDS model Classification and detection of Ransomware	RNN solves the limitation of lack of memory to recall previous events^21^	Vanishing gradient problem, Exploding gradient, Long-term dependency
LSTM	It is a DL method which is a variant of RNN architecture. The LSTM consists of gates and memory cells. The LSTM method works in three stages: Forget Gate, Update Gate/input gate, and Output Gate	Intrusion/Malware detection; Classifying malicious traffic; Detection of malicious activities; Detection of attacks in Fog-to-Things; Detecting abnormalities in the IoT; Detect SQL and XSS attacks, LSTM can be used to learn patterns and features in network data to classify them as attack or benign^21^ The LSTM can be used to recognize repeated attack patterns in a long sequence of packets^21^ The LSTM network is enormously important to detect malware injection, phishing sites	Most powerful; Higher accuracy; Well-suited for tasks which need long-term memory, Strong against the vanishing gradient problem, Able to learn patterns in long sequences^21^, It is effective in^21^ training on unstructured datasets such as those of the IoT, It perfectly classifies normal and attack instances into their respective classes^21^, It reduces the burden of feature engineering^21^ over classical ML because LSTM operates on raw data, It is resilient against adversaries^21^, Gradient vanishing or explosion problem can be solved using different gate units in LSTM; This method is also designed to solve RNN problems LSTMs have a unique formulation/construct that allows them to prevent vanilla RNN scaling and training problems, avoiding the back propagation (BP) error which either explodes or decays exponentially	More complicated
MLP	It is a type of ANN. The MLPs are trained with the BP (back-propagation) algorithm. It is a FFNN (Feedforward Neural Networks). FFNN is the first proposed NNs	DoS attack detection in sensor networks, Classify network traffic, Classification and Detection of Ransomware	MLPs solve complex problems	MLPs suffer from vanishing gradients, overfitting, and underfitting,
DBN	DBN is a generative model. It is a type of DNN^49^. It is a multi-layer belief network^13^ where each layer is RBM	Intrusion Detection^13^ Network abnormal behavior detection^49^	DBN achieved better performance than RBM model	High Computational cost
RBM	RBM is a generative model. It is a kind of ANN^49^. The learning and testing phases are the two process types that comprises. RBMs can be used within the context of IDS	Intrusion Detection	The RBM can correctly distinguish between anomalous and normal behavior within a network^9^;	High Computational cost
DAE	DAE is a generative model. It was utilized by Shone et al., 2018 for^13^ cyber security intrusion detection. It consists of an input, multiple hidden layers and output layer	IoT botnet attack detection^49^ Intrusion Detection	DAE is important for feature extraction	High computational time
GAN	It is relatively new class of ANNs^56^, the main aim of which is to generate certain objects^55^. The GAN combines two NNs, one of which generates/creates the objects, while the second estimates them	Anomaly detection, Intrusion detection^55^, Security anomalies^55^	Generation of objects from specific classes, Fast convergence^55^	Difficult to train
AAE	AAE (Adversarial Autoencoder) similar to FFNN. It is a probabilistic autoencoder which uses the GAN^58^. AAE is a generative autoencoder.	Anomaly detection	AAE increases the performance of the autoencoder with adversarial loss^58^	AAE imposes complicated distributions

**Table 6:** Advantages and disadvantages of IDS for IoT.

	Tasks	Case study	Strengths	Weaknesses
*IDS*				
NIDS	Attacks detection, Anomaly detection, Intrusion detection	Detecting attacks in IoT environments; Improving accuracy, and data integrity; Improving IoT Architecture Security	NIDS will not affect the performances of hosts^59^; Cross-platform and more scalable	Does not indicate whether the attack was successful or no^59^
HIDS			Easy to deploy; HIDS telling us if an attack is successful or no^59^	DOS attack or network scans are not detected by HIDS; HIDS breakdown if the Operating System break down by the attack^59^

As shown in Table 6, NIDSs are cross-platform and more scalable compared to HIDSs. To offer a higher level of security, these solutions can be used together. For the AI algorithms, as shown in Table 5, the results teach us that each AI algorithm has its advantages and disadvantages/limitations that could affect its performance. From the table shown in Table 5, we conclude that the LSTM has many advantages and few drawbacks compared to other AI algorithms. As synthesis, we can say that there is no automatic method to choose or to propose a model of NNs, that means the absence of a systematic method making it possible to define the best topology of the network and the number of neurons to be placed in the hidden layer (s). Otherwise, if there is no "black box" restriction; NNs will be the better choice.

There are many AI models that can be used for IoT security in classification and/or detection contexts, but each model has its own advantages, drawbacks and applicable scenario. Thereby, we should select appropriate model/classifier according to the Table 5 which give description of models, strength and weakness and application/problem in IoT security systems. The advantages and the disadvantages of the model will affect the choice of the algorithm. We often observe the use of AI algorithms with their different techniques and especially NNs. We conclude that the technique of classifying and detecting IoT attacks using LSTM is an efficient method. i.e., the choice of LSTM for IoT attack classification and detection is really a good choice. In this paper, we discuss the different types of AI, the related works of each type, aiming at the classification and detection of IoT attacks and intrusions. This study proves the effectiveness of using LSTM in the context of the classification and detection of attacks and intrusions in IoT systems. It is therefore necessary to use LSTM technique. Thus, we make a comparison of the proposed AI classification techniques with other classification technique named CTM in order to have a relevant method for classifying or/and detecting attacks in IoT systems.

The findings indicate that many research have incorporated deep learning with IoT security or machine learning with IoT security. Nevertheless, there is a lack of studies in incorporating machine learning and deep learning for IoT security. However, these investigations have proven the feasibility and efficiency of incorporating machine and deep learning for IoT security. This paper is based on two studies: (1) a performance study of artificial intelligence for IoT security; and (2) a comparative study between machine learning and deep learning for IoT security. In conclusion, the performance study has proven that LSTM is the best method compared to other traditional techniques of classification. LSTM algorithm is always the best reaching technique in these datasets BoT-IoT, AWID, Mirai-CCU, Mirai-RGU, ISCX2012, USTC-TFC2016, and CSE-CIC-IDS2018, with respectively 98%, 98.22%, 99.46%, 100%, 99.99%, 99.99%, and 98.394% compared to other AI models (between 74.78 and 99%). On the other hand, a comparative study has proven that LSTM has many benefits and few drawbacks compared to other AI classification techniques. By examining these findings, it is clear that the LSTM model is the best classifier according to its highest accuracy (100%). This means that when comparing the resulting state-of-the-art performance, LSTM achieves the best performance in all metrics especially the accuracy, with the seven-datasets. In addition, we clearly see that the LSTM deals with learning long-term dependencies, it also deals with sensor data^11^. Furthermore, the LSTM can be developed as a problem of classification in a supervised manner for use in attack detection. Moreover, an LSTM model can solve the problem of gradient vanishing or explosion. In addition, unlike traditional ML, the LSTM can be used to recognize repeated attack patterns in a long sequence of packets regardless/independent of window size^21^. Also, it is clear that the LSTM technique is resilient against adversaries since adversaries cannot fit to feature learning algorithms in order to advance their breaching techniques. Indeed, the precision-recall curve of LSTM is greater and higher which indicates that it perfectly classifies normal and attack instances into their respective classes^21^. Thus, the long memory of LSTM model over a long data sequence allowed the model/approach to perform better compared to other classical ML techniques. This means that the performance of the LSTM model leads to the efficiency of deep learning for attack classification and intrusion detection. The LSTM method is also designed to solve RNN problems. All these reasons might encourage many researchers from exploring its potential in classifying and detecting cyber security threats in IoT systems. These findings pave the way for more robust security for IoT environments. Moreover, the findings of the research on the contribution of artificial intelligence in the security of IoT systems are an indication of Deep Learning’s potential to succeed in IoT cybersecurity environment. Which confirmed the superiority of DL algorithms over traditional ML approaches.

There is a lack of appropriate and big IoT datasets that contain updated and new attacks behaviors. Therefore, researchers should work on building other IoT datasets to achieve the best possible findings/results using different deep learning techniques to do attacks classification and detection in IoT environment. This paper encourages the successful application and adoption of DL based LSTM in improving IoT security systems. Also, the use of other techniques than ML such as XAI in order to enhance and improve the security of IoT security systems is another interesting research direction.

In this section, the comparative study used can be useful for the reader to get an idea about security attacks, vulnerabilities, and a survey on AI algorithms for IoT security systems. We conducted a comparative study of artificial intelligence approaches for attacks classification and intrusion detection, we analyzed seventeen machine and deep learning approaches, including K-Nearest Neighbour, Support Vector Machine, Decision Tree, Nave Bayes, Ensemble Learning, Random Forest, Neural Network, Artificial Neural Networks, Convolutional Neural Networks, Recurrent Neural Networks, Long Short-Term Memory, Multi-layer Perceptron, Restricted Boltzmann Machine, Deep Belief Network, Deep Autoencoder, Generative Adversarial Network, Adversarial Autoencoder.

In addition, some studies proposed hybrid-based NIDS (Network Intrusion Detection Systems) for IoT systems that combines anomaly detection and signature-based detection. The anomaly detection method detects unknown or novel attacks, and the signature-based detection method detects known attacks. RPiDS proposed by^60^ which is a novel intrusion detection or IDS architecture for the IoT environment based on existing tools. Authors identified the Snort as the IDS for their system, and the Raspberry Pi as a base hardware. Snort is the widely known open-source IDS, it is also a rule/signature-based IDS, multi-platform, lightweight NIDS, but it has just a single thread. They experimented with different configurations of Snort, namely detection engine as well as number of rules loaded, to perform the on the usage of RAM and CPU rate by Snort on the Raspberry. Their experiments demonstrate that the Raspberry Pi is capable of hosting Snort, by making RPiDS approach a feasible solution. The results show that their proposed architecture can effectively serve as IDS in IoT. However, the study didn’t investigate alternatives to Snort; they didn’t consider experimenting with other open-source IDSs like Suricata. Suricata’s main appreciated feature is the multi-threading, while, Snort’s main criticized feature is the single-threading.

Recently, hybrid intrusion detection has been proposed by^47^ that combines anomaly detection and misuse detection. Hybrid detection systems train the two models (i.e., anomaly and misuse detection models) independently, then aggregate their results. Authors^47^, proposed a new hybrid/combined detection method which hierarchically integrates an anomaly detection model and a misuse detection model. Their experiment results show that their proposed method performed better than the conventional methods in detecting both known attacks and unknown attacks. In the integration process, the misuse model captures known attacks, then uses the anomaly model to supplement the ability to detect the unknown attack. First, the program used training data consisted of known attack traffic and normal traffic to train a C4.5 DT (Decision Tree) algorithm in order to build the misuse detection model, then trained 1-class SVMs (Support Vector Machine) on unknown attack sub dataset of the C4.5 DT branches to create/establish multiple anomaly detection models.

Moreover, existing efforts on network traffic anomaly detection include statistics-based methods and machine learning based methods. The proposed hybrid method was based on wavelet analysis and RVM (Relevance Vector Machine)^61^. Malicious attacks and network failures are the security problems with network. Therefore, to ensure network security, detecting anomalies of network traffic is the effective manner. For network traffic prediction, simple statistical models are not good enough^61^. For that, authors^61^ proposed a hybrid method that was a combination between statistical and ML techniques to solve the network traffic prediction problem and anomaly detection. The proposed model was composed of (1) network traffic data decomposition into low-frequency components and high-frequency components using wavelet decomposition, then, (2) non-linear model RVM (Relevance Vector Machine) and Auto Regressive Moving Average (ARMA) are employed for prediction. i.e., for prediction, they applied the RVM model on high-frequency components and ARMA model on low-frequency components. In the experiment, they used a dataset (http://newsfeed.ntcu.net/-news/2006) from the network traffic library. The dataset collected 300 network traffic data records per hour from August 1st to November 10th, 2011. 250 records were used for training and the latter 50 for testing. In their research, they used Relative Root Mean Square Error (RRMSE) and Root Mean Square Error (RMSE) to measure the prediction results. The results demonstrate the feasibility of combining machine learning methods and statistical methods together. Besides, their experiments evaluate the efficiency of their model.

In this context, integrating intrusion detection systems (IDS) into an IoT system gives better insight to secure and monitor the IoT system. Therefore, a secure architecture of IoT system is needed with recommended technologies^4,62^. Therefore, implementing an IDS on the network layer is needed. Moreover, communication technologies and protocols in IoT systems must also be secured^4^. As mentioned before, MQTT is the most widely utilized telemetry protocol in IoT^1,3,4,63^, it is one of the most popular protocols for implementing IoT networks^64^. However, the MQTT protocols do not provide the required security level by default^64^. So, evaluating the performance of the MQTT protocol^3^ on the different QoS (Quality of Services) levels without and with security implementation is required^64,65^. Montori et al.^65^ proposed an extension to the standard protocol MQTT called LA-MQTT (Location-Aware MQTT), for spatial-aware communications on IoT scenarios. LA-MQTT supports location-aware IoT communications.

The relevance of this contribution: among the critics, there are several authors working on “the detection of intrusions in an IoT system using AI”, or “the state of the art on AI for intrusion detection in the IoT”. The strength of this paper compared to these works is therefore the problem of comparison don't pose. So, it is new work with new and relevant results. There are no works on the same research question "what is the best classifier?", “which is the best AI algorithm for improving IoT security?”, “can we use the chosen algorithm in order to classify and/or detect intrusions and attacks in the IoT security systems?”, “which datasets are most suitable for IoT systems?”. Moreover, this is the strength, and the added value of this paper.

### Challenges of AI with IoT

AI and IoT have challenges. In^66^, author presented in their paper the relation of AI with IoT. When we merge the AI and IoT, the challenges of AI and IoT are listed below:

- *Security*: the important collected data must be secured;
- *Compatibility and complexity*: combining these many devices that are connected together with different technologies may cause many difficulties;
- *Artificial stupidity*: to perform basic tasks perfectly the AI program is unable; AI algorithms must be well used and developed to interpret more accurate data in order to take rational decision;
- *Lack of confidence*: business and customers have a little confidence about the integrity of data created and to protect IoT devices;
- *Cloud attacks*: large amount of data is stored in cloud, for this reason, the risk of data security increase;
- *Technology*: competition for all technologies is the biggest challenge.

## Conclusion

Security and privacy are two important factors to consider. Hence, home automation, smart hotel are some applications of AI system in the IoT^66^. This paper aimed to report some related works to IoT security in order to evaluate the performance of the AI in term of accuracy indicator, the employed methods, the used and most available IoT datasets, as well as the achieved results. Now, AI is a solution in many areas, it becomes a key factor in IoT security especially in classifying and detecting attacks. As a results, AI is showing amazing results in the field of the IoT security systems. AI fit perfectly with classifying and detecting attacks in IoT environment. This study presents the AI approaches, especially used in security to handling and improving the IoT security systems.

This survey was oriented towards AI techniques for classification and intrusion detection (the works that have been done between 2017 and 2023, the accuracy that has been deduced from the algorithms, etc.). The main goal is to focus on comparing the efficiency of the AI algorithms. This performance comparison aims us to deduce the AI algorithm used in IoT security system that gives the best prediction results. Finally, the right results got with the LSTM method. Using this method, we have proven that the LSTM is the best technique for classification compared to the other traditional methods. For classification, the noted observation is that the DL-based LSTM method has proven that it is the best method compared to the other traditional techniques. Only LSTM results outperformed other AI classifiers. Indeed, using LSTM algorithm in an intrusion detection field achieves better results than other AI algorithms. Moreover, new and big IoT-datasets should be generated with more IoT attacks, novel attack types, updated attacks behaviors in order to build the new updated AI-IDS model aiming at the elimination of false negatives and false positives.

Regarding datasets, the most recent and new IoT dataset that was created in the year 2021 is MQTTSet. While, IoT-23, TON_IoT, and MQTT-IOT-IDS2020 were created in the year 2020. Regarding AI techniques and models, the chosen algorithm demonstrates the higher accuracy (with 100% which is perfect) when compared to other Artificial Intelligence algorithms existing in the literature. It allowed a detection with lower loss rates (0.58%) and a better performance in terms of prediction time. LSTM model has the best results of (reach 100%) which that could be applied on attack classification and anomaly detection in IoT.

As a result, LSTM is the best algorithms among the AI techniques for improving the IoT security, in spite the fact that it recorded high accuracy. For this reason, the purpose of this study is to get pure results that will be used in the future work and situate our contribution among other works carried out in the same context in the literature in order to better direct the future work towards good results.

This study helps to discover a proper classification and detection algorithm (LSTM) and an IoT dataset (TON_IoT) that will be implemented in future work to approve an algorithm achieve accurate, relevant and attractive results by using an effective tool like Python, which means the chosen model will be applied on an IoT dataset. In the upcoming work, we will test on the dataset an intrusion detector based on such an algorithm to approve it and evaluate that model by estimating its performance on the test set. We have vigorously reviewed the important aspects of AI and IoT security, specifically, the current state and potential future directions.

## Data Availability

The IoT datasets analyzed during the current study as shown in Table 3 are publicly available and not on reasonable request of their corresponding author. These IoT datasets are publicly available, free, downloadable via their persistent web link to datasets as shown in Table 3.
